# Analysis of influencing factors for syphilis serofast and development and evaluation of a predictive model

**DOI:** 10.3389/fimmu.2026.1897585

**Published:** 2026-08-14

**Authors:** Jing Wen, Zhen Li, Wenchao Li, Danxuan Liu, Yongxiang Yu, Zhenzhen Wang, Gongqi Yu, Pengcheng Huai, Wen Sun, Qing Pan, Chuan Wang, Hongqing Tian, Hong Liu, Furen Zhang

**Affiliations:** 1 Dermatology Hospital of Shandong First Medical University, Jinan, Shandong, China; 2 Shandong Provincial Institute of Dermatology and Venereology, Shandong Academy of Medical Sciences, Jinan, Shandong, China

**Keywords:** influencing factors, predictive model, serofast, syphilis, toluidine red unheated serum test (TRUST)

## Abstract

**Background:**

With the rising incidence of syphilis in recent decades, the number of patients with syphilis serofast has also increased, posing challenges to clinical management of patients and increasing the social burden.

**Objective:**

In this study, we aimed to analyze influencing factors associated with syphilis serofast, develop and validate a predictive model for clinical prevention of syphilis serofast.

**Methods:**

A total of 373 syphilis patients were selected as the study subjects. Using a multivariate logistic regression model to analyze the relevant influencing factors of syphilis serofast, and the receiver operating curve (ROC) was used to evaluate the predictive performance of relevant influencing factors for syphilis serofast. Additionally, 187 syphilis patients were selected to verify the performance of the aforementioned model.

**Results:**

Among the 373 syphilis patients, serofast occurred in 134 patients (35.92%). Multivariate logistic analysis indicated that syphilis stage (SS), baseline TRUST titer (BTT), and TRUST titer changes at three months post-treatment (TTC-3M) were closely associated with the occurrence of syphilis serofast. Further ROC curve analysis revealed that the combined prediction of serofast by these three factors yielded an area under the ROC curve of 0.87 (95% CI: 0.84-0.91, P < 0.001). Subsequently, the predictive performance of the aforementioned model was evaluated, achieving an actual sensitivity of 0.88 and a specificity of 0.81 in 187 syphilis patients.

**Conclusions:**

SS, BTT, and TTC-3M are key factors influencing the occurrence of syphilis serofast. The predictive model constructed based on these three factors exhibits good efficacy and possesses clinical application value.

## 1 Introduction

Syphilis is a sexually transmitted disease caused by Treponema pallidum (T. pallidum) infection, characterized by latent infection and recurrent episodes (1, 2). According to the most recent estimate by WHO, there were approximately 17.7 million adults aged between 15 years and 49 years with syphilis globally in 2021 (3, 4). In China, the incidence of syphilis has shown an overall increasing trend. In 2019, China reported 535,819 cases of syphilis. The incidence of syphilis increased from 7.1217 per 100,000 population in 2004 to 38.3677 per 100,000 population in 2019. (5) Because there is no vaccine to prevent syphilis, control of syphilis is mainly dependent on the timely diagnosis and prompt treatment of infected individuals and their sexual partners with benzathine penicillin G (BPG), the first-line drug for all stages of syphilis (6, 7). The evaluation of treatment efficacy primarily relies on serological testing. A negative result in non-treponemal tests (NTTs) or a ≥4-fold decrease (equivalent to 2 dilution steps) in antibody titers is considered the criterion for achieving serological cure 6 months after treatment (8, 9).

“Syphilis serofast” describes a unique serological reaction in patients with syphilis. After consistent anti-syphilitic therapy, their clinical symptoms have resolved, and other systemic infections such as neurosyphilis and re-infection with syphilis have been excluded. However, the NTT remains positive for a long time (10). Approximately 35% of patients in the serofast state experience disease recurrence; increased penicillin doses or extended treatment durations typically do not reverse this condition (11). Furthermore, syphilis serofast is closely associated with neurosyphilis and involves multiple systems (12, 13). Due to the inability to predict serofast status in syphilis and take timely preventive measures, the risk of its progression to the aforementioned symptoms significantly increases (12). It is reported that the occurrence of serofast may be associated with a range of factors, including clinical correlates, immunological cytokines, and syphilis subtypes (14–17). However, since the key factors influencing syphilis serofast have not been discovered and its formation mechanism remains inadequately elucidated, there is an inability to achieve accurate prediction of syphilis serofast. With the increasing prevalence of syphilis in China, there has been a significant rise in the rate of serofast cases, posing a substantial clinical and public health challenge (10).

To investigate the key influencing factors of syphilis serofast, this study conducted a retrospective analysis of 373 syphilis patients with complete clinical records over the past decade. The aim was to identify determinants of serofast development, construct a predictive model, evaluate its performance, and ultimately achieve individualized prediction of serofast occurrence, providing an effective tool for the management of clinical patients with syphilis serofast.

## 2 Materials and methods

### 2.1 Study subjects

A retrospective study was performed in the Dermatology Hospital of Shandong First Medical University from January 2013 to June 2025. Diagnoses of syphilis patients were established in accordance with the criteria reported by *Rosanna W Peeling* et al. (1). Patients with HIV coinfection, neurosyphilis, ocular syphilis, syphilis reinfection, or incomplete clinical and follow-up records were excluded. Additionally, we screened for and eliminated potential confounders that may distort nontreponemal serology results, such as autoimmune disorders, pregnancy, acute febrile illnesses, and malignant neoplasms.

Definitions of syphilitic serofast status are provided by the 2021 US CDC, 2020 European IUSTI/EACS, 2024 UK BASHH guidelines, and the 2023 Chinese Expert Consensus (18–20). During follow-up, TRUST titers are monitored every three months in the first year and every six months in the second to third year, requiring sustained titers for over three months. The above clinical and follow-up data are all complete and valid.

The specific criteria for determining syphilis serofast are as follows:

#### 2.1.1 Clearly identify the stage and type of syphilis

Acquired syphilis is divided into early and late syphilis. Early syphilis comprises the primary, secondary and early latent stages-syphilis of less than two years from acquisition of infection. Late syphilis refers to late latent syphilis, gummatous, nervous system and cardiovascular syphilis. The diagnosis of syphilis requires comprehensive judgment, which is based on the epidemiological background, clinical manifestations, recent treatments with antibiotics, and laboratory test results, to confirm suspected or diagnosed cases. The laboratory-based diagnosis of syphilis includes the Treponema pallidum particle agglutination (TPPA), the Toluidine red unheated serum test (TRUST) (One of the commonly methods of NTT), and/or nucleic acid amplification tests (such as qPCR) (21).

##### 2.1.1.1 Primary syphilis

Primary syphilis is characterized by one or more ulcerated lesions known as chancres of syphilis at the site of initial infection. These ulcers can heal within 2–10 weeks even without treatment.

The following diagnostic tests are employed:

1. qPCR, which can directly detect *T. pallidum* from lesion samples.
2. Both TPPA and TRUST, which are negative in the early stage of primary syphilis. It takes 1–4 weeks after the appearance of the chancre for these tests to become reactive. Therefore, a negative RPR or rapid syphilis test does not rule out syphilis at the primary syphilis stage.

##### 2.1.1.2 Secondary syphilis

Secondary syphilis presents with signs of disseminated syphilis approximately 3–6 weeks after infection, though this period can extend up to six months.

The following diagnostic tests are used:

1. qPCR: *T. pallidum* can be detected by molecular methods from lesions of secondary syphilis, such as condylomata lata and mucous patches.
2. Both TPPA and TRUST are almost always reactive (positive) in secondary syphilis, usually at a high titer. A negative treponemal test at this stage of syphilis can reasonably be used to rule out syphilis if a false-negative result with nontreponemal tests (a prozone phenomenon) has been ruled out.

##### 2.1.1.3 Latent syphilis

Latent syphilis (i.e., cases lacking clinical manifestations) acquired within less than two years is referred to as early latent syphilis. An infection that has lasted for more than two years without clinical evidence of treponemal infection is referred to as late latent syphilis. All other cases of latent syphilis are classified as latent syphilis of unknown duration.

Late latent syphilis is diagnosed through serological tests. Both TPPA and TRUST are mostly reactive (positive). A negative treponemal test at this stage of infection can be regarded as sufficient to rule out the diagnosis of syphilis.

#### 2.1.2 Patients receive regular treatment.

All confirmed syphilis patients have been treated in accordance with the latest Chinese syphilis treatment guidelines, the “Guidelines for Clinical Diagnosis, Treatment, and Prevention of Sexually Transmitted Diseases (2020 Edition).” (22).

For early syphilis, such as primary, secondary, and early latent syphilis, the first-line treatment is benzathine penicillin G (BPG) 2.4 MU (intramuscular, single dose). Alternative treatments include ceftriaxone 0.5–1 g (intravenous or intramuscular, once daily for 10 days). If there is a penicillin allergy, doxycycline (100 mg, twice daily for 15 days) can be given (23).

The first-line treatment for late syphilis is BPG 2.4 MU (intramuscularly, once weekly for 3 weeks). If there is a penicillin allergy, doxycycline (100 mg, twice daily for 30 days) can be given.

#### 2.1.3 Exclude cases such as syphilis reinfection, neurosyphilis, and HIV infection.

The following situations need to be excluded:

1. Through a comprehensive inquiry into the epidemiological history, understand the syphilis infection status of the sexual partner and rule out syphilis reinfection. If the TRUST titer is detected to increase by two-fold or more, reinfection is highly suspected, and one course of retreatment should be administered.
2. Through comprehensive examinations such as neurological and cerebrospinal fluid tests, asymptomatic neurosyphilis, cardiovascular syphilis, etc., should be ruled out. Neurosyphilis is diagnosed based on clinical manifestations as well as cerebrospinal fluid (CSF) abnormalities, such as an elevated white blood cell (WBC) count, elevated CSF protein, or a reactive CSF-VDRL test.
3. Rule out HIV infection by conducting an HIV test.
4. A detailed medical history must also be obtained, as some underlying conditions may cause a false-positive reaction with NTTs, such as acute febrile illnesses, immunizations, pregnancy, and autoimmune disorders like rheumatoid arthritis and systemic lupus erythematosus. Such false positives usually have low titers of less than 1:8 (24).

#### 2.1.4 Clarify the judgment time for serofast.

During follow-up, TRUST titers are monitored every three months in the first year and every six months in the second year, with the titers required to remain stable (typically ≤1:8) for over three months.

This study was approved by the Ethics Committee of Dermatology Hospital of Shandong First Medical University.

### 2.2 Study design and methods

A retrospective analysis was conducted on the clinical data of syphilis patients spanning the past decade. This study collected comprehensive demographic information, including gender, age, and marital status, along with clinical diagnoses, treatment regimens, syphilis serological laboratory test results, and routine follow-up data. First, statistically analyze the seroreversion rate and time to seroreversion for syphilis patients at various stages, the incidence of serofast status, and the final TRUST titer levels. Subsequently, univariate chi-square tests and multivariate logistic regression analysis were employed to assess the correlation between the occurrence of serofast status and various factors, including patient gender, age, syphilis stage (SS), clinical treatment regimen, pretreatment baseline TRUST titer (BTT), and the magnitude of change in TRUST titer at three months post-treatment compared to pre-treatment (TTC-3M), to identify potential significant associated factors. Furthermore, an receiver operating characteristic (ROC) curve analysis was conducted to evaluate the efficacy of significant associated factors, both individually and in combination, for predicting serofast status in syphilis. A predictive model for the occurrence of serofast status was constructed by developing a nomogram. The predictive model was evaluated using data from an additional 187 syphilis patients, including basic demographic information, clinical diagnosis, laboratory test results, and follow-up information.

### 2.3 Statistical methods

Statistical analysis was performed using SPSS version 22.0 and R version 4.4.2 software. Univariate chi-square (*χ²)* tests, multivariate logistic regression analysis, and ROC curve analysis were employed. For the construction of the predictive model, the statistical approaches included RStudio, version 4.3.2 (R Foundation for Statistical Computing), with the ‘pROC’, ‘rms’, ‘ggplot2’, and ‘caret’ packages utilized to support model establishment and result visualization. Data that conformed to a normal distribution are presented as mean ± standard deviation ( $\overset{-}{x}$ ± SD), and differences between groups were compared using the independent-samples *t*-test. Count data are expressed as n (%), and differences between groups were assessed using *χ²* tests, with *P* < 0.05 indicating statistical significance.

## 3 Results

### 3.1 General patient data

**Figure 1:**
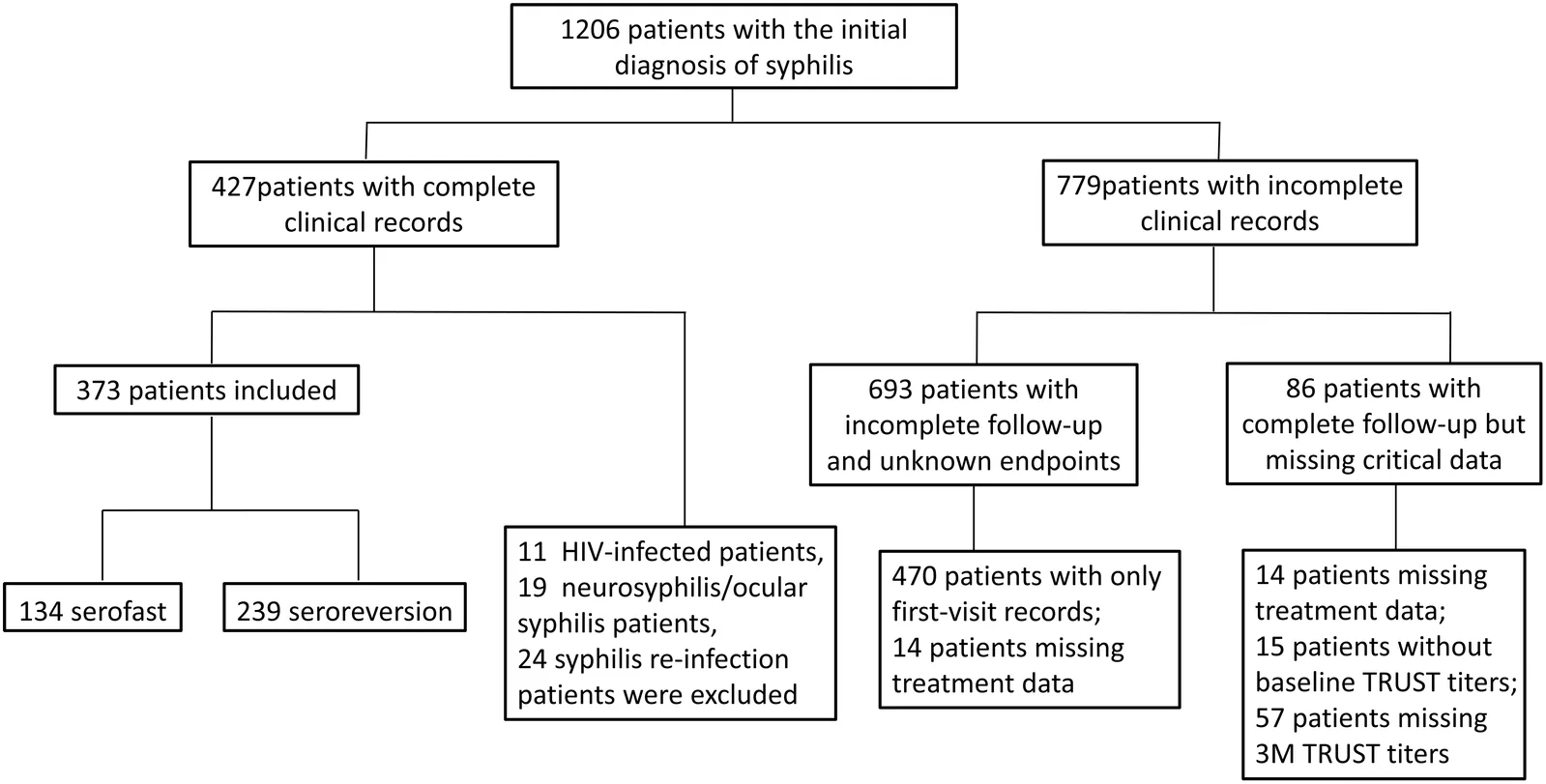
Sample selection flowchart of the study.

A total of 1206 patients diagnosed with syphilis were enrolled in this study. Among them, 693 patients lost to follow-up, 86 patients with missing clinical and follow-up data, 11 HIV-coinfected patients, 19 patients with neurosyphilis or ocular syphilis, and 24 patients with syphilis reinfection were excluded. Ultimately, a total of 373 treatment-naive syphilis patients with complete clinical data were included in the final study cohort (**Figure 1**). This group included 221 males and 152 females, with a male-to-female ratio of approximately 1.5:1. The ages ranged from 1 to 79 years, with a mean age of 45.0 ± 14.1 years. Furthermore, we collected individual baseline data for all 779 patients excluded from the primary analysis and performed comparative analyses against the 373 enrolled participants. There were no statistically significant intergroup differences with respect to sex, age strata, syphilis stage, treatment regimen, and baseline TRUST titer.

### 3.2 Seroreversion and serofast status of non-treponemal tests in syphilis patients

The cohort consisted of 373 patients with syphilis, comprising 67 cases (17.96%) of primary syphilis, 152 cases (40.75%) of secondary syphilis, and 154 cases (41.29%) of latent syphilis, including 38 cases of early latent syphilis, 70 cases of late latent syphilis, and 46 cases of irregular (with uncertain infection time) latent syphilis. With a BTT ranging from 1:1 to 1:2048, 239 patients (64.08%) experienced seroreversion within 36 months post-standardized treatment and biennial follow-up, whereas 134 (35.92%) developed a serofast status (66 males, 68 females; mean age 55.0 ± 20.5 years). Stage-specific serofast rates were 4.48% for primary, 28.29% for secondary, and 57.14% for latent syphilis, with detailed seroreversion and serofast rates by stage presented in **Table 1** and **Supplementary Table 1**. The differences in TRUST titer seroreversion rates and serofast status incidence among patients with various stages of syphilis were statistically significant (P<0.05).

**Table 1:** Seroreversion and serofast status in 373 syphilis patients [n (%)].

Diagnosis	n	Primary syphilis	Secondary syphilis	Latent syphilis		
				Early	Late	Unknown
Seroreversion	239 (64.08)	64 (95.52)	109 (71.71)	22 (57.89)	26 (37.14)	18 (39.13)
Serofast	134 (35.92)	3 (4.48)	43 (28.29)	16 (42.11)	44 (62.86)	28 (60.87)
Total	373	67	152	38	70	46

As shown in **Table 2**, changes in TRUST titers following 3 months of standard treatment were assessed. Among the 134 serofast patients, only 26.12% (35 cases) achieved a TRUST titer of ≤1:2. By contrast, 66.95% (160 cases) of the 239 seroreverters reached a TRUST titer of ≤1:2. Further analysis at 6 months demonstrated that 87.87% (210 cases) of seroreverters achieved a TRUST titer of ≤1:2, compared with only 38.81% (52 cases) of serofast patients.

**Table 2:** Comparison of TRUST titers within 6 months in 373 syphilis patients [n (%)].

Diagnosis	n	Stage		TRUST titers								
			Negative	1:1	1:2	1:4	1:8	1:16	1:32	1:64	1:128	≥1:256
Serofast	134	0M	0 (0.00)	3 (2.24)	17 (12.69)	13 (9.70)	13 (9.70)	28 (20.90)	26 (19.40)	24 (17.91)	6 (4.48)	4 (2.99)
	134	3M	0 (0.00)	10 (7.46)	25 (18.66)	22 (16.42)	36 (26.87)	24 (17.91)	13 (9.70)	2 (1.49)	0 (0.00)	2 (1.49)
	134	6M	0 (0.00)	25 (18.66)	27 (20.15)	28 (20.90)	32 (23.88)	16 (11.94)	4 (2.99)	2 (1.49)	0 (0.00)	0 (0.00)
Seroreversion	239	0M	0 (0.00)	32 (13.39)	32 (13.39)	15 (6.28)	27 (11.30)	41 (17.15)	52 (21.76)	35 (14.64)	4 (1.67)	1 (0.42)
	239	3M	69 (28.87)	49 (20.50)	42 (17.57)	40 (16.74)	23 (9.62)	13 (5.44)	3 (1.26)	0 (0.00)	0 (0.00)	0 (0.00)
	239	6M	141 (59.00)	43 (17.99)	26 (10.88)	16 (6.69)	6 (2.51)	5 (2.09)	1 (0.42)	1 (0.42)	0 (0.00)	0 (0.00)

Among the 239 syphilis patients who experienced seroreversion, 66 (27.62%) had their TRUST titers revert within three months. A total of 141 patients (59.00%) achieved seroreversion within six months, with 49 cases of primary syphilis (76.56%), 49 cases of secondary syphilis (44.95%), and 43 cases of latent syphilis (65.15%) among them. Of the 134 patients with a serofast status, 110 (82.09%) had a final serofast titer of ≤1:4, and 126 (94.03%) had a final serofast titer of ≤1:8. Specific details are provided in **Table 3**.

**Table 3:** Final TRUST status of 373 syphilis patients [n (%)].

Syphilis stage	n	Time to seroreversion for 239 seroreverters						Final TRSUT titer for 134 serofast patients					
		n	≤3 months	4~6 months	7~12 months	13~24 months	24 months	n	1:1	1:2	1:4	1:8	≥1:16
Primary syphilis	67	64	28 (43.75)	21 (32.81)	11 (17.19)	3 (4.69)	1 (1.56)	3	2 (66.67)	0 (0.00)	0 (0.00)	1 (33.33)	0 (0.00)
Secondary syphilis	152	109	12 (11.01)	37 (33.94)	28 (25.69)	22 (20.18)	10 (9.17)	43	18 (41.86)	7 (16.28)	10 (23.26)	4 (9.30)	4 (9.30)
Latent syphilis	154	66	26 (39.39)	17 (25.76)	6 (9.09)	6 (9.09)	11 (16.67)	88	29 (32.95)	19 (21.59)	25 (28.41)	11 (12.50)	4 (4.55)
Total	373	239	66 (27.62)	75 (31.38)	45 (18.83)	31 (12.97)	22 (9.21)	134	49 (36.57)	26 (19.40)	35 (26.12)	16 (11.94)	8 (5.97)

### 3.3 Analysis of influencing factors for syphilis serofast

#### 3.3.1 Univariate analysis of different influencing factors

Univariate analysis revealed statistically significant associations (P<0.05) between serofast status and gender, age (in years), clinical treatment regimen, and BTT. Notably, particularly strong correlations (P<0.001) were observed for SS and TTC-3M. Specific analysis details are presented in **Table 4**.

**Table 4:** Analysis of influencing factors for serofast in syphilis patients (Univariate and multivariate logistic regression).

Influencing factors	Syphilis patients (n)	Serofast cases (n)	Serofast rate (%)	Univariate χ² analysis		Multivariate logistic regression analysis				
				*χ²* value	*β* value	*β* value	*SE*	Wald *χ²* value	*P* value	*OR* value (95%CI)
Gender				8.812	0.003	0.129	0.288	0.200	0.655	1.137(0.647~1.998)
Male	221	66	29.86%							
Female	152	68	44.74%							
Age (years)				2.888	0.014	-0.099	0.114	0.752	0.386	0.906(0.724~1.133)
≤20	35	10	28.57%							
21~30	139	58	41.73%							
31~40	90	21	23.33%							
41~50	60	23	38.33%							
51~60	35	13	37.14%							
>60	14	9	64.29%							
SS				37.420	<0.001	0.759	0.146	27.001	<0.001	2.136(1.604~2.843)
Primary syphilis	67	3	4.48%							
Secondary syphilis	152	43	28.29%							
Latent syphilis	154	88	57.14%							
BTT				2.451	0.010	-0.738	0.097	58.409	<0.001	0.478(0.396~0.578)
1:1+	35	3	8.57%							
1:2+	49	17	34.69%							
1:4+	28	13	46.43%							
1:8+	40	13	32.50%							
1:16+	69	28	40.58%							
1:32+	78	26	33.33%							
1:64+	59	24	40.68%							
1:128+	10	6	60.00%							
≥1:256+	5	4	80.00%							
Clinical treatment regimen				6.032	0.003	0.308	0.192	2.559	0.110	1.360(0.933~1.983)
Penicillin	275	87	31.64%							
Penicillin+Other	37	22	59.46%							
Other drugs	61	25	40.98%							
TTC-3M				9.376	<0.001	1.038	0.143	52.423	<0.001	2.823(2.132~3.739)
+2	1	1	100.00%							
+1	5	5	100.00%							
0	50	36	72.00%							
-1	88	39	44.32%							
-2	87	26	29.89%							
-3	64	16	25.00%							
-4	43	8	18.60%							
-5	26	3	11.54%							
-6	9	0	0.00%							
Constant						-3.763	0.866	18.882	0.000	0.023

#### 3.3.2 Multivariate analysis of different influencing factors

Utilizing the occurrence of serofast status (0=no, 1=yes) as the dependent variable, predictors such as gender, age (in years), SS, clinical treatment regimen, BTT, and the TTC-3M were employed as independent variables for multivariate logistic regression analysis. The findings indicated that SS (*P* < 0.001, OR: 1.604-2.843), BTT (*P* < 0.001, OR: 0.396-0.578), and TTC-3M (*P* < 0.001, OR: 2.132-3.739) demonstrated statistically significant differences within the regression model. Notably, SS and TTC-3M were identified as risk factors for serofast status in syphilis patients, whereas BTT served as a protective factor. Detailed analysis results and assignment methods are presented in **Tables 4**, **5**.

**Table 5:** Variable assignment for logistic regression analysis.

Factors		Variable level assignment
Dependent variable	Serofast	No=0, Yes=1
Independent variables	Gender	Male=1, Female=2
	Age(years)	≤20 = 1, 21~30 = 2, 31~40 = 3, 41~50 = 4, 51~60 = 5, >60 = 6
	SS	Primary syphilis=1, Secondary syphilis=2, Latent syphilis=4
	BTT	Logarithmic value (0~11)
	Clinical treatment regimen	Penicillin=1, Penicillin+Other=2, Other drugs=3
	TTC-3M	-6~+2

1. Assignment explanation for TTC-3M. e.g., if BTT = 1:16, and TT-3M=1:2, then TTC-3M= -3 (a decrease of 3 dilutions, e.g., 16->8->4->2).

2. Other drugs include Ceftriaxone and Doxycycline.

#### 3.3.3 ROC Curve Analysis​

**Figure 2:**
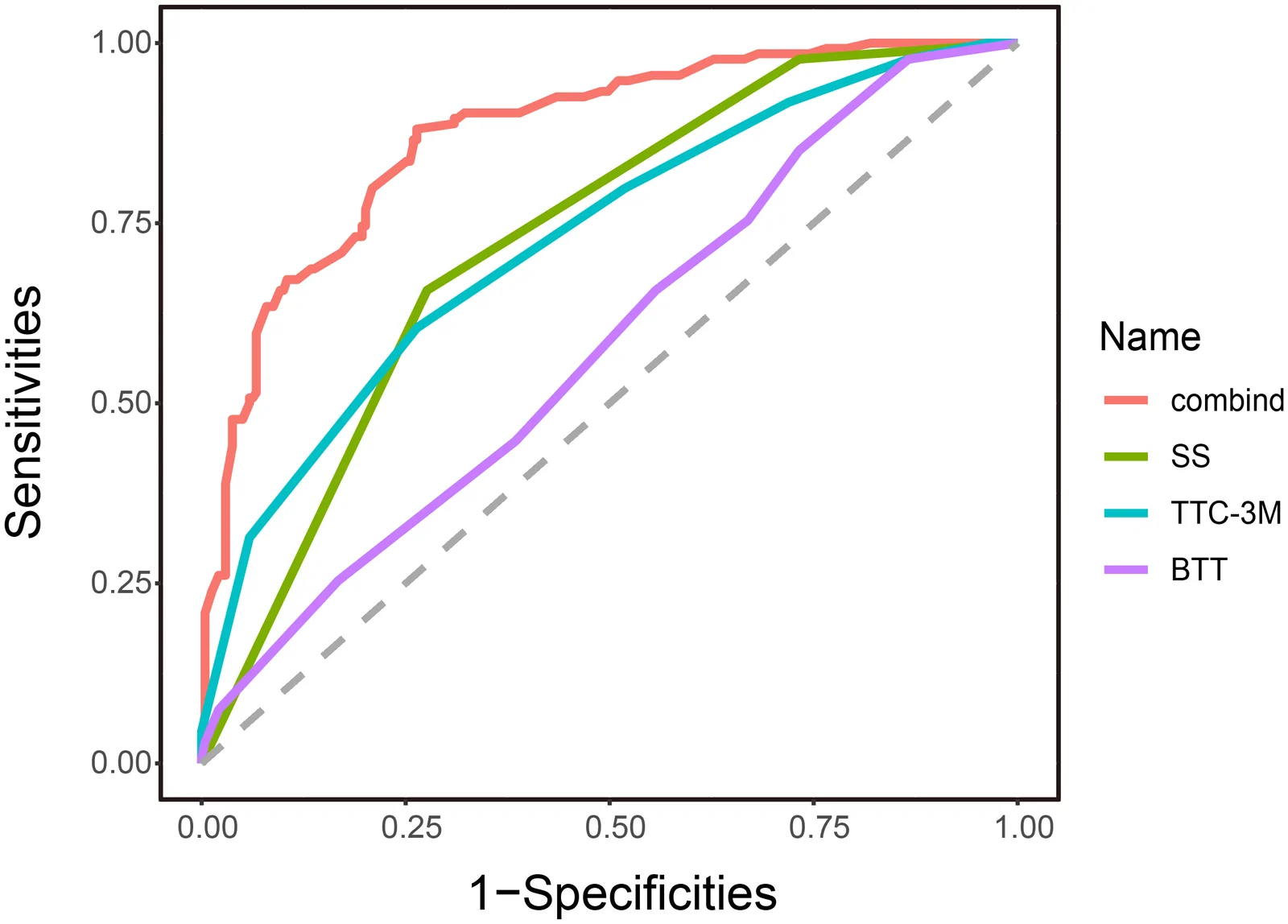
ROC curve of combined SS, BTT, and TTC-3M for predicting Syphilis serofast status.

**Figure 3:**
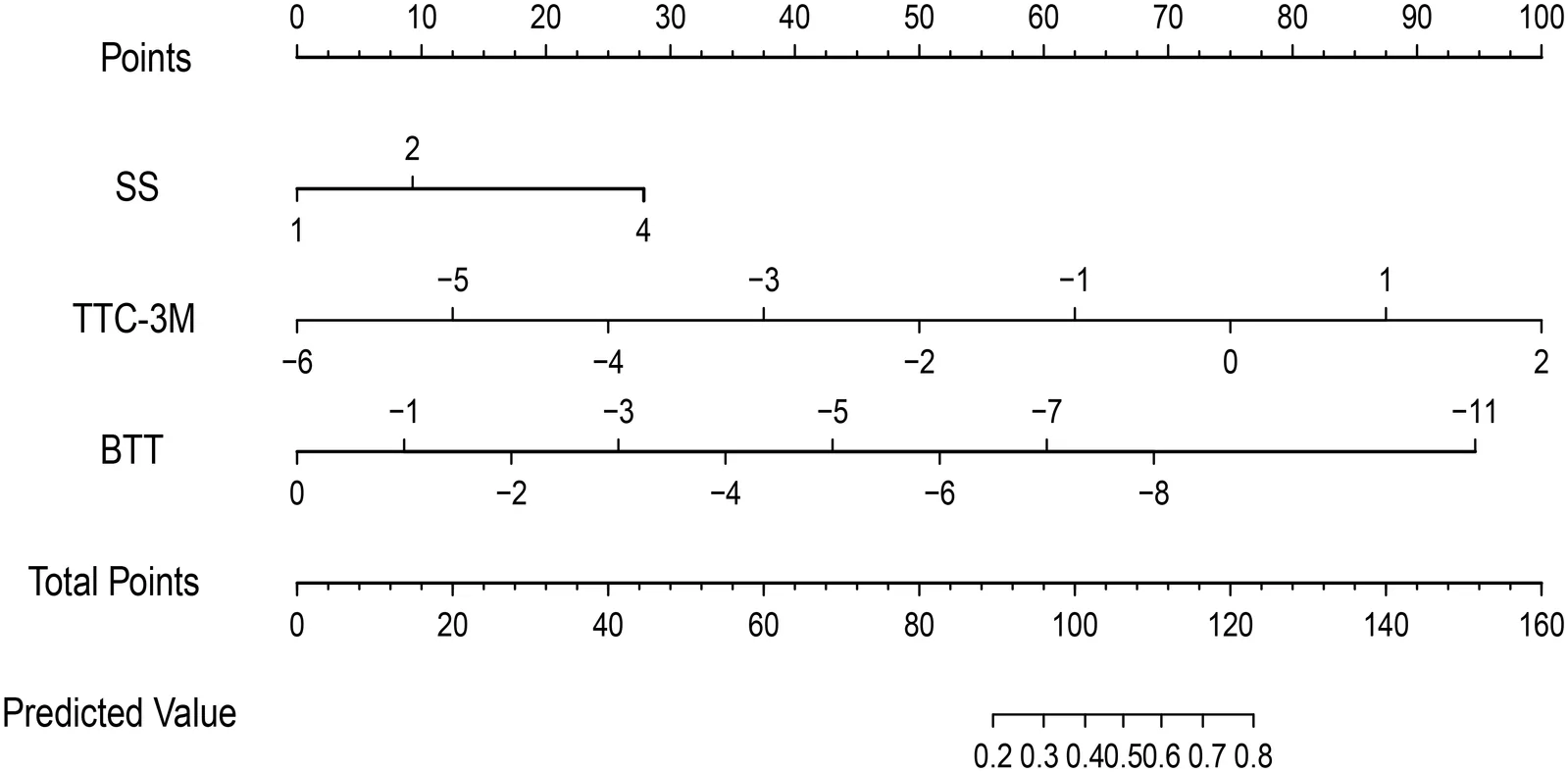
Nomogram for predicting syphilis serofast. Visual representation of the nomogram. The top row displays the point allocation for each predictor variable. Points are assigned for each level of SS, BTT, TTC-3M. Total points correspond to a predicted probability on the bottom scale.

To further evaluate the association between SS, BTT, and the TTC-3M with the occurrence of serofast status, we conducted ROC curve analysis (**Figure 2**). The results in **Table 6** indicated that the area under the ROC curve (AUC) for the combined prediction model (SS, BTT and TTC-3M) was 0.87 (95% CI: 0.84-0.91, *P* < 0.001), which was more effective than individual predictions. The calibration intercept (-3.7×10E-11; 95% CI: -0.28 - 0.28) and calibration slope (1.00; 95% CI: 0.79 - 1.21) were close to the ideal values of 0 and 1, respectively, indicating good apparent calibration of the model. Meanwhile, We verified the calibration performance of the model via internal validation with 1,000 bootstrap resamples and Hosmer-Lemeshow goodness-of-fit tests. The optimal cut-off value was determined to be 0.27, with a sensitivity of 0.88 and a specificity of 0.74. Based on these significant indicators, we further constructed a nomogram to simplify the practical prediction of serofast status (**Figure 3**). In the nomogram, the overall probability of serofast development can be predicted by summing the individual point scores corresponding to SS, BTT, and TTC-3M. For illustrative purposes, we assume a representative patient presenting with secondary syphilis, a BTT titer of 1:16, and a TTC-3M value of -4 (a 4-fold dilution decrease). According to **Table 5**, secondary syphilis is assigned a coding value of 2, which corresponds to 10 points on the **Figure 2** nomogram. Similarly, the log-transformed value of the 1:16 BTT titer equals -4, yielding 35 points in the same nomogram. Furthermore, a TTC-3M value of -4 corresponds to 25 points in **Figure 2**. The sum of these three indicators yields a total score of 70 on the **Figure 2** nomogram, corresponding to an overall predicted serofast probability below 20%.

**Table 6:** Logistic regression model prediction for syphilis serofast.

Influencing factors	AUC	*SE*	*P* value	95% CI
SS	0.73	0.024	0.000	0.68~0.77
BTT	0.58	0.030	0.038	0.52~0.64
TTC-3M	0.73	0.027	0.000	0.68~0.78
Combined prediction probability	0.87	0.019	0.000	0.84~0.91

### 3.4 Validation of the serofast prediction model​

In this study, based on the previously established nomogram for predicting serofast status in syphilis, we further included 187 additional syphilis patients from our outpatient department. Clinical parameters (SS, BTT, TTC-3M) were entered into the nomogram to generate predicted probabilities of serofast status, which were then compared with the actual outcomes (details are provided in **Table 7**). The AUC in the internal validation dataset was 0.936 (95% CI: 0.902-0.969). The validation results revealed that the predictive model achieved a sensitivity of 0.88 and a specificity of 0.81, indicating alignment between the theoretical predictive performance reflected by the ROC curve and the actual clinical validation outcomes.

**Table 7:** Predictive performance evaluation of syphilis serofast model.

		Observed cases of syphilis serofast (n)		Sensitivity (%)	Specificity (%)
		0	1		
Projected cases of syphilis serofast (n)	0	82	10	0.88	0.81
	1	19	76		

0=No serofast status; 1=Serofast status.

## 4 Discussion

Syphilis serofast refers to a unique serological phenomenon (25, 26). Insufficient understanding and diagnostic difficulties regarding syphilitic serofast status pose challenges to clinical management of patients and greatly jeopardize patients’ overall physical and mental well-being. Timely prediction and intervention constitute an effective strategy to address the aforementioned issues. However, to date, no reliable approaches are available for predicting serofast status among syphilis patients. In present study, we analyzed and identified SS, BTT, and TTC-3M as critical influencing factors for the development of syphilis serofast, based on which we constructed a predictive model, thereby providing evidence support for the clinical management of syphilis.

Previous studies have shown varying serofast incidence rates among syphilis stages: primary (ranging from 3.80% to 15.20%), secondary (11.64%-35.80%), tertiary (45.02%-45.90%), and latent syphilis (27.41%-40.50%) (14, 27). Tertiary and latent syphilis may be risk factors for serofast status (28). Another study indicates that 25%-40% of untreated secondary syphilis patients may develop neurosyphilis, which is also a risk factor for serofast (29). This study included 373 treatment-naive syphilis patients with complete data, comprising 67 primary syphilis cases (17.96%), 152 secondary syphilis cases (40.75%), and 154 latent syphilis cases (41.29%). After standardized anti-syphilitic treatment and a complete 2-year follow-up period, approximately 64.08% of patients achieved TRUST titer seroreversion without clinical relapse. Among these, 210 patients (87.87%) showed TRUST titers decreasing to (less than or equal to) 1:2 within 6 months and 141 patients (59.00%) completely seroreverted within 6 months. The remaining 134 patients developed serofast (35.92% incidence), which is consistent with previous findings. By stage, latent syphilis had the highest serofast rate (57.14%), followed by secondary (28.29%) and primary syphilis (4.48%).

NTTs, including rapid plasma reagin test (RPR) and TRUST, serve as crucial indicators for evaluating the efficacy of syphilis treatment, with NTT antibody titers closely correlating with disease activity and immune responses. A prospective study of 517 early syphilis patients (30) found that patients with higher RPR titers had lower serofast rates, and RPR titers ≥1:32 were significantly associated with serological cure-consistent with our findings. This may reflect more effective inflammatory and immune responses against *Treponema pallidum* (*T. pallidum*) in high-titer patients, predisposing them to better treatment outcomes. A key challenge in serofast determination lies in accurately staging syphilis, as treatment protocols and prognostic criteria vary by disease stage/type, while disease duration significantly impacts NTT seroreversion. To address these issues, we incorporated TTC-3M parameter, a dynamic NTT monitoring concept that eliminates uncertainties in estimating infection duration and enables reliable assessment of treatment response.

Univariate analysis revealed statistically significant associations between serofast status and gender, age (in years), clinical treatment regimen, BTT, SS and TTC-3M. A study of 1,327 HIV-negative syphilis patients (14) identified age and gender as risk factors for serofast status patients over 40 years and female patients showed higher serofast rates, consistent with our findings. This may be potentially due to immunosenescence, a higher comorbidity burden, and poorer treatment adherence in elderly populations (31). Physiological immune differences between genders and frequently subtle genital lesions in females (leading to diagnostic delays) may explain the higher serofast rates in women. Varied treatment regimens contribute to disparate therapeutic outcomes. For primary syphilis, doxycycline yields marginally lower cure rates relative to penicillin, yet this disparity lacks statistical significance. Conversely, doxycycline is associated with poorer rates of lesion resolution in patients with late or syphilis of unknown duration (32). Penicillin has remained the cornerstone of syphilis therapy since its widespread clinical uptake in the late 1940s, per prior literature. While no randomized controlled trials have formally validated its therapeutic efficacy, penicillin markedly outperforms all historical anti-syphilitic agents, and no cases of Treponema pallidum penicillin resistance have ever been documented (18). Earlier clinical data further demonstrate that escalated penicillin dosages fail to substantially alter the proportion of patients developing serofast status post-treatment (33).

In our study, Multivariate stepwise regression analysis further identified SS, BTT, and TTC-3M as independent predictors of serofast status. We found that 53.13% of primary syphilis cases seroreverted within three months post-treatment, significantly higher than secondary (11.01%) and latent syphilis (39.39%) cases. This disparity likely reflects the difference between early-stage localized infection (primary/early secondary) and disseminated disease in latent cases (34), where conventional treatment is less effective, though confounding factors cannot be excluded. Research indicates that syphilis stage and baseline RPR titer are associated with serological cure at 6 months after early syphilis treatment (35). Participants with elevated baseline titers exhibited a higher probability of attaining serological cure, a finding concordant with our results (35, 36). This observation aligns well with the results reported by Baker-Zander et al. (37), who found that rabbits immunized with VDRL antigen acquired partial protective immunity against secondary reinfection by T. pallidum. The authors proposed that elevated VDRL antibody titers could facilitate infection containment and accelerate treponemal clearance in patients with early-stage syphilis. If this mechanism holds true, elevated baseline nontreponemal titers may reflect a robust, favorable inflammatory and immune response directed against T. pallidum. Further investigations are essential to elucidate the biological basis for the serofast state and to determine whether serofast patients should undergo continued serological monitoring, retreatment, or cerebrospinal fluid examination for T. pallidum involvement. There are limited studies investigating the association between TTC-3M and the risk of syphilis serofast. Study has demonstrated that TTC-3M can serve as an early warning for the risk of serofast; patients with insufficient titer decline are unlikely to achieve sustained titer reduction during the entire follow-up period (14). In this study, we confirmed that TTC-3M is significantly associated with the development of serofast, incorporated it into the prediction model, and clarified its risk contribution to serofast.

Accurate identification of syphilis serofast is clinically critical for distinguishing between overtreatment and undertreatment (27). Our findings provide insights into the incidence and risk factors of serofast status. Meanwhile, we developed a predictive model to estimate the risk of serofast development in patients with well-documented treatment histories-namely those with definitive clinical staging who receive standardized anti-syphilis therapy and routine serial monitoring of TRUST titers. By incorporating clinical staging, titer fluctuations and other core follow-up markers, this prediction model allows personalized evaluation of serofast risk at the 3-month post-treatment time point, which provides vital guidance for refined clinical intervention. Specifically, this model facilitates the rapid identification of high-risk patients, thereby allowing for stratified management. Clinically, enhanced monitoring and early intervention strategies are implemented for high-risk individuals, which effectively reduces the risk of treatment interruption.

Notably, the predictive model provides an objective and reliable basis for clinical decision-making, guiding the formulation of personalized follow-up plans. This individualized approach not only improves patient compliance but also ensures that clinical interventions are targeted and evidence-based. Collectively, these efforts not only optimize the management process but also promote a shift from passive treatment to proactive prevention, thereby enhancing the overall quality of clinical care and providing robust support for the clinical management of syphilis.

Our study is not without its limitations. Firstly, the sample size utilized for model construction and performance assessment was insufficient, and only single-center internal validation was conducted, restricting the generalizability of this predictive model. Accordingly, further validation incorporating larger cohorts of patients from multiple centers is required to improve the model’s generalizability in future studies. Secondly, our model acts as an early post-treatment risk stratification tool instead of a baseline prognostic prediction model that can be utilized prior to treatment. Its inability to predict the risk of serofast before therapy may impairs its clinical applicability. Furthermore, as treatment outcome data were unavailable for 693 patients, residual selection bias arising from unmeasured confounders or loss to follow-up cannot be fully excluded, which may limit the generalizability of our findings. In addition, we noticed that the AUC of the internal validation cohort (0.936) was markedly higher than that of the training cohort (0.87), which may be attributed to random variation arising from the smaller sample size of the validation group.

In summary, we identified SS, BTT, and TTC-3M as key risk factors for syphilis serofast status in this study and further developed a predictive model for early post-treatment risk stratification of syphilis serofast. Recognition of individuals at high risk of serofast status enables early intervention and personalized follow-up regimens for syphilis patients, providing a valuable tool to support clinical syphilis management.

## Data Availability

The original contributions presented in the study are included in the article/**Supplementary Material**. Further inquiries can be directed to the corresponding author.

## References

[1] Peeling RW Mabey D Chen XS Garcia PJ . Syphilis. Lancet. (2023) 402:336–46. doi: 10.1016/S0140-6736(22)02348-0 37481272

[2] Peeling RW Hook EW 3rd . The pathogenesis of syphilis: the great mimicker, revisited. J Pathol. (2006) 208:224–32. doi: 10.1002/path.1903 16362988

[3] World Health Organization . Web Annex 1. Key data at a glance, in: Global progress report on HIV, viral hepatitis and sexually transmitted infections, 2021. Accountability for the global health sector strategies 2016-2021: actions for impact (2021). Geneva: World Health Organization. Available online at: http://apps.who.int/iris/bitstream/handle/10665/342808/9789240030985-eng.pdf (Accessed September 17, 2022).

[4] Jane R Stephen VH Eline K Nicola L Magnus U Laith JAR . Chlamydia, gonorrhoea, trichomoniasis and syphilis: global prevalence and incidence estimates, 2016. Bull World Health Organ. (2019) 97:548–62. doi: 10.2471/BLT.18.228486 31384073 PMC6653813

[5] Li JY Yang YC Huang BR Zeng JQ . Epidemiological characteristics of syphilis in mainland China, 2004 to 2019. J Int Med Res. (2024) 52:3000605241258465. doi: 10.1177/03000605241258465 38886868 PMC11184993

[6] Rocha AFB Araújo MAL Taylor MM Kara EO Broutet NJN . Treatment administered to newborns with congenital syphilis during a penicillin shortage in 2015, Fortaleza, Brazil. BMC Pediatr. (2021) 21:166. doi: 10.1186/s12887-021-02619-x 33832443 PMC8028238

[7] Stephen NF Melanie MT Margaret S Maeve BM Sanni S Manuel L . Shortages of benzathine penicillin for prevention of mother-to-child transmission of syphilis: an evaluation from multi-country surveys and stakeholder interviews. PloS Med. (2017) 14:e1002473. doi: 10.1371/journal.pmed.1002473 29281619 PMC5744908

[8] Ferris S Stephanie H Maxwell B Pirathaban S Robert N . The laboratory diagnosis of syphilis. J Clin Microbiol. (2021) 59:e0010021. doi: 10.1128/JCM.00100-21 33980644 PMC8451404

[9] World Health Organization . Laboratory diagnosis of sexually transmitted infections, including human immunodeficiency virus. Geneva: World Health Organization (2013). Available online at: https://wkc.who.int/resources/publications/i/item/9789241505840#:~:text (Accessed April 15, 2026).

[10] Committee on Sexually Transmitted Diseases, China Dermatologist Association . Expert consensus on clinical management of serofast state in syphilis. Chin J Dermatol. (2023) 56:383–8. doi: 10.35541/cjd.20220656

[11] Clement ME Okeke NL Hicks CB . Treatment of syphilis: a systematic review. JAMA. (2014) 312:1905–17. doi: 10.1001/jama.2014.13259 25387188 PMC6690208

[12] Cai SN Long J Chen C Wan G Lun WH . Incidence of asymptomatic neurosyphilis in serofast Chinese syphilis patients. Sci Rep. (2017) 7:15456. doi: 10.1038/s41598-017-15641-w 29133821 PMC5684362

[13] Zhou PY Gu X Lu HK Guan ZF Qian YH . Re-evaluation of serological criteria for early syphilis treatment efficacy: progression to neurosyphilis despite therapy. Sex Transm Infect. (2012) 88:342–5. doi: 10.1136/sextrans-2011-050247 22363023

[14] Tong ML Lin LR Liu GL Zhang HL Zeng YL Zheng WH . Factors associated with serological cure and the serofast state of HIV-negative patients with primary, secondary, latent, and tertiary syphilis. PloS One. (2013) 8:e70102. doi: 10.1371/journal.pone.0070102 23894598 PMC3720935

[15] Lin LR Tong ML Fu ZG Dan B Zheng WH Zhang CG . Evaluation of a colloidal gold immunochromatography assay in the detection of Treponema pallidum specific IgM antibody in syphilis serofast reaction patients: a serologic marker for the relapse and infection of syphilis. Diagn Microbiol Infect Dis. (2011) 70:10–6. doi: 10.1016/j.diagmicrobio.2010.11.015 21388769

[16] Wu YN Lu LF Song XJ Liu XS Yang Y Chen L . Clinical and immunological characteristics of HIV/syphilis co-infected patients following long-term antiretroviral treatment. Front Public Health. (2024) 11:1327896. doi: 10.3389/fpubh.2023.1327896 38288435 PMC10823526

[17] Paul G Wesselmann J Adzic D Malin JJ Suarez I Priesner V . Predictors of serofast state after treatment for early syphilis in HIV-infected patients. HIV Med. (2021) 22:165–71. doi: 10.1111/hiv.12985 33128333

[18] Workowski KA Bachmann LH Chan PA Johnston CM Muzny CA Park I . Sexually transmitted infections treatment guidelines, 2021. MMWR Recomm Rep. (2021) 70:1–187. doi: 10.15585/mmwr.rr7004a1 34292926 PMC8344968

[19] Janier M Unemo M Dupin N Tiplica GS Potocnik M Patel R . 2020 European guideline on the management of syphilis. J Eur Acad Dermatol Venereol. (2021) 35:574–88. doi: 10.1111/jdv.16946 33094521

[20] Kingston M Apea V Evans C Fifer H Foster K Patrick P . BASHH UK guidelines for the management of syphilis 2024. Int J STD AIDS. (2024) 35:1142–60. doi: 10.1177/09564624241280406 39270129

[21] National Center for STD Control, Chinese Center for Disease Control and Prevention; Society of Dermatology and Venereology, Chinese Medical Association; Subcommittee on Sexually Transmitted Diseases, Chinese Society of Dermatology . Guidelines for the diagnosis and treatment of syphilis, gonorrhea and genital chlamydia trachomatis infection. Chin J Dermatol. (2020) 53:168–79. doi: 10.35541/cjd.20190808

[22] Wang QQ . Guidelines for Clinical Diagnosis, Treatment and Prevention of Sexually Transmitted Diseases (2nd Edition). Shanghai: Shanghai Scientific & Technical Publishers (2020) p. 1–178.

[23] Markewitz R Pauli D Wandinger KP . The modern epidemic of syphilis. N Engl J Med. (2020) 382:2379–80. doi: 10.1056/NEJMra1901593 32521147

[24] Leiva A Shaw M Paine K Manneh K McAdam K Mayaud P . Management of sexually transmitted diseases in urban pharmacies in The Gambia. Int J STD AIDS. (2001) 12:444–52. doi: 10.1258/0956462011923471 11394980

[25] Martyna K Konrad K Maciej P . Serofast state after syphilis treatment: implications and recommendations for clinical practice. Narrative review. Postepy Dermatol Alergol. (2025) 42:215–20. doi: 10.5114/ada.2024.147332 40672722 PMC12262024

[26] Darina C Marie Z Chen L Petra P Michal S Qin X . Whole genome sequences of three Treponema pallidum ssp. pertenue strains: yaws and syphilis treponemes differ in less than 0.2% of the genome sequence. PloS NeglTrop Dis. (2012) 6:e1471. doi: 10.1371/journal.pntd.0001471 22292095 PMC3265458

[27] Cao Q Li Y Hu YB He BS Tang Y Cao T . Serofast status in syphilis: Pathogenesis to therapeutics. Clin Chim Acta. (2024) 560:119754. doi: 10.1016/j.cca.2024.119754 38815665

[28] Lin LR Zheng WH Tong ML Fu ZG Liu GL Fu JG . Further evaluation of the characteristics of Treponema pallidum-specific IgM antibody in syphilis serofast reaction patients. Diagn Microbiol Infect Dis. (2011) 71:201–7. doi: 10.1016/j.diagmicrobio.2011.07.005 21899981

[29] Mehrabian S Raycheva MR Petrova EP Tsankov NK Traykov LD . Neurosyphilis presenting with dementia, chronic chorioretinitis and adverse reactions to treatment: a case report. cases J. (2009) 2:8334. doi: 10.4076/1757-1626-2-8334 19918420 PMC2769430

[30] Zhang RL Wang QQ Zhang JP Yang LJ . Molecular subtyping of Treponema pallidum and associated factors of serofast status in early syphilis patients: Identified novel genotype and cytokine marker. PloS One. (2017) 12:e0175477. doi: 10.1371/journal.pone.0175477 28410389 PMC5391950

[31] Corsenac P Noël M Rouchon B Hoy D Roth A . Prevalence and sociodemographic risk factors of chlamydia, gonorrhoea and syphilis: a national multicentre STI survey in New Caledonia, 2012. BMJ Open. (2015) 5:e007691. doi: 10.1136/bmjopen-2015-007691 26353867 PMC4567678

[32] Zengarini C Carpanese MA Vara G Conni A Piraccini BM Gaspari V . Analysis of serological treatment response to doxycycline versus benzathine penicillin in syphilis infections, a retrospective single-center study. Dermatol Ther. (2022) 35:e15586. doi: 10.1111/dth.15586 35594004 PMC9540744

[33] Schroeter AL Lucas JB Price EV Falcone VH . Treatment for early syphilis and reactivity of serologic tests. JAMA. (1972) 221:471–6. doi: 10.1001/jama.1972.03200180015003 4556863

[34] Qin J Yang T Wang H Feng T Liu X . Potential Predictors for Serofast State after Treatment among HIV-Negative Persons with Syphilis in China: A Systematic Review and Meta-Analysis. Iran J Public Health. (2015) 44:155–69. doi: 10.18502/ijph.v44i2.224 PMC4401873 25905049

[35] Arlene CS Mark W David HM Frieda B Kathleen VD Peter L . Predictors of serological cure and Serofast State after treatment in HIV-negative persons with early syphilis. Clin Infect Dis. (2011) 53:1092–9. doi: 10.1093/cid/cir671 21998287 PMC3205200

[36] Zeng X Ouyang Y Wang H Liu L Chen J Zhu C . Risk factors of serofast state in patients undergoing syphilis: a meta-analysis of 17 cohort studies. Front Immunol. (2025) 16:1689904. doi: 10.3389/fimmu.2025.1689904 41425592 PMC12714923

[37] Baker-Zander SA Shaffer JM Lukehart SA . VDRL antibodies enhance phagocytosis of Treponema pallidum by macrophages. J Infect Dis. (1993) 167:1100–5. doi: 10.1093/infdis/167.5.1100 8486943

