# Supplementary material for: Analysis of influencing factors for syphilis serofast and development and evaluation of a predictive model
Source: Front Immunol. 2026 Aug 14;17:1897585. doi: 10.3389/fimmu.2026.1897585 (PMC13521870; doi:10.3389/fimmu.2026.1897585)
Supplement: Supplementary file 1 [file Table1.docx]

***Supplementary Material***

Title

Analysis of influencing factors for syphilis serofast and development and evaluation of a predictive model

**Authors**:

Jing Wen^1,2#^, Zhen Li^1,2#^, Wenchao Li^1,2^, Danxuan Liu^1,2^, Yongxiang Yu^1,2^, Zhenzhen Wang^1,2^, Gongqi Yu^1,2^, Pengcheng Huai^1,2^, Wen Sun^1,2^, Qing Pan^1,2^, Chuan Wang^1,2*^, Hongqing Tian^1,2^, Hong Liu^1,2^, Furen Zhang ^1,2^

^1^Dermatology Hospital of Shandong First Medical University, 250022, Jinan, Shandong, China.

^2^Shandong Provincial Institute of Dermatology and Venereology, Shandong Academy of Medical Sciences, 250022, Jinan, Shandong, China.

^#^These authors contributed equally to this work.

***** **Corresponding author**:

Prof. Chuan Wang

Dermatology Hospital of Shandong First Medical University, Jinan, Shandong, China.

Shandong Provincial Institute of Dermatology and Venereology, Shandong Academy of Medical Sciences, Jinan, Shandong, China.

27397 Jingshi Lu, Jinan 250022, Shandong Province, P.R. China

Tel: 86-0531-87298848

Fax: 86-0531-87984734

E-mail: wangchuan86@126.com

Table S1 Clinical characteristics of syphilis patients for predictive model establishment

| No. | Serofast(0=No; 1=Yes) | Gender  (1=Male; 2=Female) | Age(years)  (≤20=1; 21~30=2; 31~40=3; 41~50=4; 51~60=5; >60=6) | SS (Primary syphilis=1; Secondary syphilis=2; Tertiary syphilis=3; Latent syphilis=4) | Clinical treatment regimen  (Penicillin=1; Penicillin+Other=2; Other drugs=3) | BTT | Logarithmic value of BTT | TT-3M | TTC-3M | TT-6M | TT-12M | final TT | Time to seroreversion |
| --- | --- | --- | --- | --- | --- | --- | --- | --- | --- | --- | --- | --- | --- |
| 1 | 1 | 1 | 5 | 4 | 1 | 1:8+ | -3 | 1:8+ | 0 | 1:8+ | 1:4+ | 1:4+ |  |
| 2 | 0 | 1 | 2 | 4 | 2 | 1:64+ | -6 | 1:4+ | -4 | N |  |  | 4~6M- |
| 3 | 0 | 1 | 1 | 4 | 1 | 1:2+ | -1 | N | -2 | N |  |  | ≤3M- |
| 4 | 1 | 2 | 2 | 4 | 1 | 1:2+ | -1 | 1:2+ | 0 | 1:1+ | 1:1+ | 1:1+ |  |
| 5 | 0 | 2 | 2 | 1 | 1 | 1:32+ | -5 | N | -6 | N |  |  | ≤3M- |
| 6 | 0 | 1 | 3 | 1 | 3 | 1:8+ | -3 | 1:2+ | -2 | N |  |  | 4~6M- |
| 7 | 1 | 1 | 4 | 4 | 3 | 1:2+ | -1 | 1:2+ | 0 | 1:2+ | 1:1+ | 1:1+ |  |
| 8 | 0 | 1 | 2 | 4 | 3 | 1:8+ | -3 | 1:4+ | -1 | 1:4+ |  |  | 13~24M- |
| 9 | 1 | 2 | 3 | 4 | 2 | 1:1+ | 0 | 1:1+ | 0 | 1:1+ | 1:1+ | 1:1+ |  |
| 10 | 1 | 2 | 2 | 4 | 1 | 1:2+ | -1 | 1:2+ | 0 | 1:2+ | 1:2+ | 1:1+ |  |
| 11 | 1 | 1 | 2 | 2 | 3 | 1:2+ | -1 | 1:2+ | 0 | 1:2+ | 1:2+ | 1:1+ |  |
| 12 | 0 | 2 | 2 | 4 | 1 | 1:1+ | 0 | 1:1+ | 0 | N |  |  | 4~6M- |
| 13 | 0 | 2 | 2 | 4 | 1 | 1:8+ | -3 | 1:1+ | -3 | N |  |  | 4~6M- |
| 14 | 1 | 1 | 2 | 4 | 1 | 1:2+ | -1 | 1:2+ | 0 | 1:1+ | 1:1+ | 1:1+ |  |
| 15 | 0 | 2 | 5 | 4 | 3 | 1:1+ | 0 | N | -1 | N |  |  | ≤3M- |
| 16 | 0 | 2 | 4 | 2 | 1 | 1:64+ | -6 | 1:8+ | -3 | 1:1+ |  |  | 7~12M- |
| 17 | 0 | 1 | 3 | 1 | 1 | 1:8+ | -3 | 1:1+ | -3 | 1:1+ |  |  | 7~12M- |
| 18 | 0 | 1 | 3 | 1 | 3 | 1:2+ | -1 | 1:1+ | -1 | N |  |  | 4~6M- |
| 19 | 1 | 2 | 6 | 4 | 3 | 1:16+ | -4 | 1:8+ | -1 | 1:8+ | 1:16+ | ≥1:16+ |  |
| 20 | 1 | 1 | 3 | 4 | 3 | 1:2+ | -1 | 1:2+ | 0 | 1:1+ | 1:1+ | 1:1+ |  |
| 21 | 1 | 2 | 3 | 4 | 3 | 1:32+ | -5 | 1:16+ | -1 | 1:2+ | 1:2+ | 1:2+ |  |
| 22 | 0 | 1 | 3 | 2 | 1 | 1:64+ | -6 | 1:8+ | -3 | 1:1+ |  |  | 13~24M- |
| 23 | 1 | 2 | 6 | 4 | 1 | 1:64+ | -6 | 1:32+ | -1 | 1:4+ | 1:4+ | 1:1+ |  |
| 24 | 1 | 2 | 2 | 4 | 1 | 1:4+ | -2 | 1:8+ | 1 | 1:2+ | 1:4+ | 1:1+ |  |
| 25 | 1 | 2 | 4 | 4 | 3 | 1:4+ | -2 | 1:4+ | 0 | 1:2+ | 1:2+ | 1:2+ |  |
| 26 | 1 | 2 | 2 | 4 | 1 | 1:4+ | -2 | 1:4+ | 0 | 1:1+ | 1:1+ | 1:1+ |  |
| 27 | 0 | 2 | 4 | 2 | 2 | 1:16+ | -4 | 1:1+ | -4 | 1:64+ |  |  | ＞24M- |
| 28 | 0 | 2 | 2 | 2 | 1 | 1:32+ | -5 | 1:2+ | -4 | 1:1+ |  |  | ＞24M- |
| 29 | 0 | 2 | 5 | 4 | 1 | 1:64+ | -6 | 1:32+ | -1 | 1:2+ |  |  | ＞24M- |
| 30 | 0 | 2 | 1 | 4 | 1 | 1:1+ | 0 | N | -1 | N |  |  | ≤3M- |
| 31 | 0 | 2 | 2 | 2 | 3 | 1:8+ | -3 | 1:4+ | -1 | 1:2+ |  |  | 13~24M- |
| 32 | 0 | 1 | 2 | 2 | 1 | 1:16+ | -4 | 1:2+ | -3 | N |  |  | 4~6M- |
| 33 | 0 | 2 | 4 | 2 | 1 | 1:64+ | -6 | 1:8+ | -3 | N |  |  | 4~6M- |
| 34 | 1 | 1 | 2 | 1 | 1 | 1:16+ | -4 | 1:4+ | -2 | 1:1+ | 1:1+ | 1:1+ |  |
| 35 | 1 | 2 | 2 | 4 | 3 | 1:16+ | -4 | 1:16+ | 0 | 1:8+ | 1:4+ | 1:2+ |  |
| 36 | 0 | 1 | 3 | 2 | 3 | 1:32+ | -5 | 1:4+ | -3 | 1:4+ |  |  | ＞24M- |
| 37 | 0 | 2 | 2 | 1 | 1 | 1:8+ | -3 | 1:1+ | -3 | 1:1+ |  |  | 7~12M- |
| 38 | 0 | 1 | 2 | 4 | 1 | 1:4+ | -2 | 1:1+ | -2 | N |  |  | 4~6M- |
| 39 | 1 | 2 | 2 | 4 | 3 | 1:4+ | -2 | 1:4+ | 0 | 1:2+ | 1:2+ | 1:2+ |  |
| 40 | 1 | 1 | 2 | 4 | 2 | 1:64+ | -6 | 1:32+ | -1 | 1:16+ | 1:16+ | 1:8+ |  |
| 41 | 1 | 2 | 2 | 4 | 1 | 1:2+ | -1 | 1:1+ | -1 | 1:2+ | 1:1+ | 1:1+ |  |
| 42 | 1 | 2 | 3 | 4 | 1 | 1:32+ | -5 | 1:16+ | -1 | 1:16+ | 1:8+ | 1:4+ |  |
| 43 | 1 | 2 | 6 | 4 | 3 | 1:2+ | -1 | 1:1+ | -1 | 1:1+ | 1:4+ | 1:4+ |  |
| 44 | 1 | 1 | 2 | 4 | 1 | 1:16+ | -4 | 1:8+ | -1 | 1:4+ | 1:4+ | 1:1+ |  |
| 45 | 0 | 2 | 3 | 4 | 2 | 1:8+ | -3 | N | -4 | N |  |  | ≤3M- |
| 46 | 1 | 1 | 4 | 2 | 1 | 1:64+ | -6 | 1:8+ | -3 | 1:2+ | 1:1+ | 1:1+ |  |
| 47 | 0 | 1 | 2 | 2 | 2 | 1:32+ | -5 | 1:2+ | -4 | N |  |  | 4~6M- |
| 48 | 1 | 2 | 2 | 4 | 3 | 1:32+ | -5 | 1:8+ | -2 | 1:4+ | 1:1+ | 1:1+ |  |
| 49 | 1 | 2 | 2 | 2 | 1 | 1:16+ | -4 | 1:2+ | -3 | 1:1+ | 1:1+ | 1:1+ |  |
| 50 | 1 | 1 | 2 | 2 | 2 | 1:64+ | -6 | 1:2+ | -5 | 1:4+ | 1:4+ | 1:4+ |  |
| 51 | 0 | 1 | 4 | 4 | 1 | 1:2+ | -1 | N | -2 | N |  |  | ≤3M- |
| 52 | 1 | 1 | 2 | 4 | 3 | 1:16+ | -4 | 1:16+ | 0 | 1:8+ | 1:4+ | 1:2+ |  |
| 53 | 1 | 2 | 2 | 4 | 1 | 1:32+ | -5 | 1:32+ | 0 | 1:8+ | 1:8+ | 1:8+ |  |
| 54 | 0 | 2 | 1 | 2 | 1 | 1:32+ | -5 | 1:4+ | -3 | 1:4+ |  |  | ＞24M- |
| 55 | 0 | 2 | 5 | 4 | 1 | 1:1+ | 0 | N | -1 | N |  |  | 4~6M- |
| 56 | 0 | 2 | 1 | 1 | 1 | 1:128+ | -7 | 1:16+ | -3 | 1:4+ |  |  | 13~24M- |
| 57 | 1 | 1 | 1 | 2 | 2 | 1:16+ | -4 | 1:16+ | 0 | 1:2+ | 1:2+ | 1:2+ |  |
| 58 | 0 | 2 | 2 | 4 | 1 | 1:1+ | 0 | N | -1 | 1:1+ |  |  | 7~12M- |
| 59 | 1 | 1 | 4 | 2 | 2 | 1:256+ | -8 | 1:32+ | -3 | 1:8+ | 1:4+ | 1:4+ |  |
| 60 | 1 | 1 | 5 | 4 | 3 | 1:2+ | -1 | 1:2+ | 0 | 1:1+ | 1:1+ | 1:1+ |  |
| 61 | 1 | 2 | 1 | 4 | 1 | 1:128+ | -7 | 1:16+ | -3 | 1:16+ | 1:4+ | 1:2+ |  |
| 6 | 0 | 1 | 3 | 1 | 1 | 1:4+ | -2 | 1:1+ | -2 | N |  |  | 4~6M- |
| 63 | 0 | 1 | 2 | 1 | 1 | 1:8+ | -3 | 1:2+ | -2 | N |  |  | 4~6M- |
| 64 | 1 | 1 | 2 | 4 | 1 | 1:32+ | -5 | 1:16+ | -1 | 1:4+ | 1:4+ | 1:2+ |  |
| 65 | 0 | 1 | 2 | 2 | 1 | 1:32+ | -5 | 1:2+ | -4 | N |  |  | 4~6M- |
| 66 | 0 | 1 | 1 | 1 | 1 | 1:16+ | -4 | 1:2+ | -3 | 1:1+ |  |  | 7~12M- |
| 67 | 0 | 2 | 3 | 4 | 3 | 1:2+ | -1 | 1:1+ | -1 | N |  |  | 4~6M- |
| 68 | 1 | 1 | 3 | 4 | 2 | 1:4+ | -2 | 1:2+ | -1 | 1:1+ | 1:4+ | 1:4+ |  |
| 69 | 1 | 1 | 1 | 2 | 1 | 1:16+ | -4 | 1:4+ | -2 | 1:1+ | 1:1+ | 1:1+ |  |
| 70 | 1 | 2 | 1 | 4 | 1 | 1:32+ | -5 | 1:8+ | -2 | 1:2+ | 1:1+ | 1:1+ |  |
| 71 | 0 | 2 | 3 | 4 | 3 | 1:1+ | 0 | 1:1+ | 0 | N |  |  | 4~6M- |
| 72 | 0 | 2 | 2 | 1 | 1 | 1:16+ | -4 | 1:4+ | -2 | 1:1+ |  |  | 7~12M- |
| 73 | 0 | 1 | 1 | 4 | 1 | 1:1+ | 0 | 1:1+ | 0 | 1:1+ |  |  | 13~24M- |
| 74 | 0 | 1 | 4 | 1 | 3 | 1:4+ | -2 | N | -3 | N |  |  | ≤3M- |
| 75 | 0 | 2 | 3 | 2 | 2 | 1:64+ | -6 | 1:4+ | -4 | 1:1+ |  |  | 7~12M- |
| 76 | 0 | 1 | 3 | 2 | 1 | 1:64+ | -6 | 1:4+ | -4 | 1:1+ |  |  | 7~12M- |
| 77 | 0 | 2 | 2 | 4 | 1 | 1:8+ | -3 | 1:2+ | -2 | N |  |  | 4~6M- |
| 78 | 1 | 1 | 2 | 4 | 1 | 1:8+ | -3 | 1:2+ | -2 | 1:2+ | 1:2+ | 1:1+ |  |
| 79 | 0 | 1 | 4 | 1 | 1 | 1:2+ | -1 | 1:1+ | -1 | N |  |  | 4~6M- |
| 80 | 0 | 2 | 2 | 4 | 1 | 1:16+ | -4 | 1:4+ | -2 | 1:4+ |  |  | 13~24M- |
| 81 | 1 | 2 | 1 | 2 | 2 | 1:64+ | -6 | 1:16+ | -2 | 1:4+ | 1:2+ | 1:1+ |  |
| 82 | 0 | 1 | 2 | 2 | 1 | 1:64+ | -6 | 1:8+ | -3 | 1:8+ |  |  | ＞24M- |
| 83 | 0 | 2 | 3 | 2 | 1 | 1:32+ | -5 | 1:4+ | -3 | 1:1+ |  |  | 7~12M- |
| 84 | 1 | 2 | 4 | 4 | 1 | 1:8+ | -3 | 1:4+ | -1 | 1:2+ | 1:2+ | 1:1+ |  |
| 85 | 0 | 1 | 4 | 2 | 3 | 1:16+ | -4 | 1:8+ | -1 | 1:2+ |  |  | 7~12M- |
| 86 | 1 | 2 | 3 | 4 | 1 | 1:8+ | -3 | 1:2+ | -2 | 1:2+ | 1:4+ | 1:4+ |  |
| 87 | 1 | 2 | 2 | 4 | 1 | 1:32+ | -5 | 1:32+ | 0 | 1:8+ | 1:8+ | 1:4+ |  |
| 88 | 0 | 1 | 1 | 2 | 1 | 1:64+ | -6 | 1:2+ | -5 | 1:2+ |  |  | ＞24M- |
| 89 | 1 | 2 | 1 | 2 | 3 | 1:64+ | -6 | 1:32+ | -1 | 1:16+ | 1:16+ | ≥1:16+ |  |
| 90 | 0 | 2 | 2 | 2 | 3 | 1:1+ | 0 | N | -1 | N |  |  | 4~6M- |
| 91 | 0 | 1 | 2 | 1 | 2 | 1:4+ | -2 | 1:1+ | -2 | N |  |  | 4~6M- |
| 92 | 0 | 2 | 4 | 4 | 3 | 1:1+ | 0 | N | -1 | N |  |  | ≤3M- |
| 93 | 0 | 1 | 4 | 4 | 1 | 1:1+ | 0 | N | -1 | N |  |  | ≤3M- |
| 94 | 1 | 1 | 6 | 4 | 2 | 1:4+ | -2 | 1:8+ | 1 | 1:8+ | 1:8+ | 1:8+ |  |
| 95 | 0 | 2 | 3 | 4 | 2 | 1:64+ | -6 | 1:8+ | -3 | 1:1+ |  |  | 13~24M- |
| 96 | 0 | 1 | 3 | 2 | 1 | 1:32+ | -5 | 1:8+ | -2 | 1:16+ |  |  | 13~24M- |
| 97 | 0 | 1 | 1 | 2 | 1 | 1:16+ | -4 | 1:4+ | -2 | 1:4+ |  |  | 7~12M- |
| 98 | 1 | 1 | 3 | 4 | 3 | 1:32+ | -5 | 1:8+ | -2 | 1:8+ | 1:8+ | 1:4+ |  |
| 99 | 1 | 2 | 3 | 4 | 1 | 1:64+ | -6 | 1:16+ | -2 | 1:16+ | 1:16+ | 1:4+ |  |
| 100 | 1 | 2 | 2 | 4 | 1 | 1:8+ | -3 | 1:4+ | -1 | 1:4+ | 1:4+ | 1:1+ |  |
| 101 | 0 | 2 | 3 | 4 | 1 | 1:32+ | -5 | 1:4+ | -3 | 1:4+ |  |  | ＞24M- |
| 102 | 1 | 1 | 3 | 2 | 2 | 1:64+ | -6 | 1:8+ | -3 | 1:2+ | 1:2+ | 1:1+ |  |
| 103 | 1 | 2 | 2 | 4 | 1 | 1:16+ | -4 | 1:8+ | -1 | 1:8+ | 1:8+ | 1:8+ |  |
| 104 | 0 | 1 | 5 | 1 | 1 | 1:2+ | -1 | N | -2 | N |  |  | ≤3M- |
| 105 | 1 | 2 | 2 | 4 | 3 | 1:16+ | -4 | 1:2+ | -3 | 1:2+ | 1:8+ | 1:8+ |  |
| 106 | 0 | 2 | 3 | 2 | 1 | 1:16+ | -4 | 1:4+ | -2 | 1:1+ |  |  | 13~24M- |
| 107 | 0 | 1 | 3 | 4 | 1 | 1:1+ | 0 | N | -1 | N |  |  | ≤3M- |
| 108 | 0 | 2 | 3 | 1 | 1 | 1:32+ | -5 | 1:2+ | -4 | 1:1+ |  |  | 13~24M- |
| 109 | 0 | 2 | 5 | 4 | 3 | 1:1+ | 0 | N | -1 | N |  |  | ≤3M- |
| 110 | 0 | 1 | 3 | 4 | 1 | 1:8+ | -3 | N | -4 | N |  |  | ≤3M- |
| 111 | 1 | 2 | 1 | 2 | 1 | 1:64+ | -6 | 1:32+ | -1 | 1:32+ | 1:8+ | 1:1+ |  |
| 112 | 0 | 1 | 3 | 2 | 3 | 1:16+ | -4 | 1:2+ | -3 | 1:1+ |  |  | ＞24M- |
| 113 | 1 | 1 | 2 | 2 | 1 | 1:32+ | -5 | 1:8+ | -2 | 1:4+ | 1:4+ | 1:1+ |  |
| 114 | 1 | 2 | 2 | 4 | 2 | 1:16+ | -4 | 1:4+ | -2 | 1:4+ | 1:4+ | 1:1+ |  |
| 115 | 1 | 1 | 2 | 4 | 1 | 1:16+ | -4 | 1:8+ | -1 | 1:2+ | 1:2+ | 1:2+ |  |
| 116 | 0 | 1 | 4 | 1 | 3 | 1:16+ | -4 | 1:1+ | -4 | N |  |  | 4~6M- |
| 117 | 1 | 1 | 3 | 2 | 2 | 1:16+ | -4 | 1:8+ | -1 | 1:2+ | 1:1+ | 1:1+ |  |
| 118 | 1 | 1 | 2 | 2 | 2 | 1:64+ | -6 | 1:8+ | -3 | 1:4+ | 1:8+ | 1:1+ |  |
| 119 | 0 | 1 | 5 | 1 | 1 | 1:2+ | -1 | 1:1+ | -1 | N |  |  | 4~6M- |
| 120 | 0 | 1 | 3 | 4 | 1 | 1:4+ | -2 | 1:2+ | -1 | 1:2+ |  |  | ＞24M- |
| 121 | 0 | 1 | 5 | 1 | 1 | 1:4+ | -2 | N | -3 | N |  |  | ≤3M- |
| 122 | 0 | 2 | 3 | 4 | 3 | 1:2+ | -1 | N | -2 | N |  |  | ≤3M- |
| 123 | 0 | 2 | 2 | 2 | 3 | 1:32+ | -5 | 1:4+ | -3 | 1:4+ |  |  | 13~24M- |
| 124 | 1 | 2 | 2 | 4 | 1 | 1:32+ | -5 | 1:8+ | -2 | 1:8+ | 1:8+ | 1:8+ |  |
| 125 | 0 | 1 | 2 | 2 | 3 | 1:4+ | -2 | 1:1+ | -2 | 1:1+ |  |  | 7~12M- |
| 126 | 1 | 2 | 2 | 4 | 3 | 1:1+ | 0 | 1:1+ | 0 | 1:1+ | 1:1+ | 1:1+ |  |
| 127 | 1 | 1 | 3 | 4 | 1 | 1:8+ | -3 | 1:8+ | 0 | 1:4+ | 1:4+ | 1:2+ |  |
| 128 | 1 | 2 | 2 | 4 | 3 | 1:8+ | -3 | 1:8+ | 0 | 1:4+ | 1:4+ | 1:4+ |  |
| 129 | 0 | 1 | 2 | 1 | 3 | 1:2+ | -1 | N | -2 | N |  |  | ≤3M- |
| 130 | 0 | 2 | 3 | 2 | 1 | 1:64+ | -6 | 1:8+ | -3 | 1:1+ |  |  | 7~12M- |
| 131 | 1 | 2 | 2 | 2 | 2 | 1:64+ | -6 | 1:8+ | -3 | 1:8+ | 1:8+ | 1:1+ |  |
| 132 | 0 | 2 | 3 | 2 | 1 | 1:32+ | -5 | N | -6 | N |  |  | ≤3M- |
| 133 | 0 | 2 | 1 | 2 | 1 | 1:32+ | -5 | 1:16+ | -1 | 1:16+ |  |  | 13~24M- |
| 134 | 0 | 1 | 4 | 4 | 1 | 1:8+ | -3 | 1:2+ | -2 | 1:1+ |  |  | 7~12M- |
| 135 | 1 | 2 | 2 | 4 | 1 | 1:2+ | -1 | 1:2+ | 0 | 1:2+ | 1:2+ | 1:2+ |  |
| 136 | 1 | 2 | 5 | 4 | 1 | 1:256+ | -8 | 1:32+ | -3 | 1:16+ | 1:8+ | 1:4+ |  |
| 137 | 1 | 2 | 4 | 4 | 3 | 1:2+ | -1 | 1:2+ | 0 | 1:1+ | 1:1+ | 1:1+ |  |
| 138 | 1 | 1 | 5 | 2 | 1 | 1:32+ | -5 | 1:4+ | -3 | 1:1+ | 1:1+ | 1:1+ |  |
| 139 | 0 | 1 | 3 | 1 | 1 | 1:2+ | -1 | 1:1+ | -1 | N |  |  | 4~6M- |
| 140 | 0 | 1 | 3 | 2 | 1 | 1:32+ | -5 | 1:4+ | -3 | 1:1+ |  |  | 7~12M- |
| 141 | 0 | 2 | 2 | 2 | 1 | 1:32+ | -5 | 1:4+ | -3 | N |  |  | 4~6M- |
| 142 | 0 | 1 | 2 | 2 | 1 | 1:128+ | -7 | 1:4+ | -5 | 1:2+ |  |  | 13~24M- |
| 143 | 1 | 2 | 2 | 4 | 1 | 1:16+ | -4 | 1:16+ | 0 | 1:4+ | 1:8+ | 1:8+ |  |
| 144 | 0 | 1 | 2 | 1 | 1 | 1:2+ | -1 | N | -2 | N |  |  | ≤3M- |
| 145 | 1 | 2 | 2 | 2 | 2 | 1:128+ | -7 | 1:8+ | -4 | 1:1+ | 1:1+ | 1:1+ |  |
| 146 | 1 | 1 | 4 | 4 | 1 | 1:2+ | -1 | 1:1+ | -1 | 1:1+ | 1:2+ | 1:2+ |  |
| 147 | 0 | 2 | 4 | 2 | 1 | 1:32+ | -5 | N | -6 | N |  |  | ≤3M- |
| 148 | 0 | 1 | 2 | 1 | 1 | 1:16+ | -4 | 1:4+ | -2 | 1:1+ |  |  | 13~24M- |
| 149 | 0 | 1 | 5 | 2 | 1 | 1:32+ | -5 | 1:2+ | -4 | 1:2+ |  |  | 13~24M- |
| 150 | 0 | 1 | 2 | 2 | 1 | 1:128+ | -7 | 1:2+ | -6 | 1:1+ |  |  | 7~12M- |
| 151 | 0 | 1 | 2 | 2 | 1 | 1:64+ | -6 | 1:4+ | -4 | N |  |  | 4~6M- |
| 152 | 1 | 2 | 2 | 4 | 1 | 1:64+ | -6 | 1:32+ | -1 | 1:8+ | 1:8+ | 1:4+ |  |
| 153 | 0 | 1 | 1 | 4 | 1 | 1:64+ | -6 | 1:16+ | -2 | 1:2+ |  |  | 13~24M- |
| 154 | 1 | 1 | 4 | 4 | 3 | 1:2+ | -1 | 1:1+ | -1 | 1:2+ | 1:2+ | 1:2+ |  |
| 155 | 1 | 2 | 2 | 4 | 1 | 1:16+ | -4 | 1:8+ | -1 | 1:8+ | 1:4+ | 1:2+ |  |
| 156 | 0 | 2 | 2 | 4 | 1 | 1:64+ | -6 | 1:8+ | -3 | 1:8+ |  |  | ＞24M- |
| 157 | 1 | 1 | 5 | 2 | 1 | 1:2048+ | -11 | 1:256+ | -3 | 1:64+ | 1:64+ | ≥1:16+ |  |
| 158 | 0 | 2 | 2 | 4 | 1 | 1:8+ | -3 | 1:8+ | 0 | N |  |  | 4~6M- |
| 159 | 0 | 2 | 2 | 4 | 1 | 1:64+ | -6 | 1:8+ | -3 | N |  |  | 4~6M- |
| 160 | 1 | 1 | 3 | 4 | 1 | 1:128+ | -7 | 1:32+ | -2 | 1:32+ | 1:8+ | 1:1+ |  |
| 161 | 0 | 1 | 1 | 2 | 1 | 1:64+ | -6 | 1:8+ | -3 | 1:2+ |  |  | 13~24M- |
| 162 | 0 | 2 | 3 | 2 | 3 | 1:32+ | -5 | 1:4+ | -3 | 1:8+ |  |  | 13~24M- |
| 163 | 0 | 1 | 3 | 4 | 3 | 1:2+ | -1 | 1:1+ | -1 | 1:4+ |  |  | ＞24M- |
| 164 | 0 | 1 | 2 | 4 | 1 | 1:32+ | -5 | 1:8+ | -2 | 1:2+ |  |  | ＞24M- |
| 165 | 0 | 2 | 2 | 2 | 1 | 1:16+ | -4 | 1:4+ | -2 | N |  |  | 4~6M- |
| 166 | 0 | 2 | 4 | 2 | 1 | 1:64+ | -6 | 1:8+ | -3 | 1:1+ |  |  | 13~24M- |
| 167 | 1 | 1 | 4 | 1 | 1 | 1:128+ | -7 | 1:64+ | -1 | 1:4+ | 1:16+ | 1:8+ |  |
| 168 | 1 | 2 | 6 | 4 | 3 | 1:4+ | -2 | 1:2+ | -1 | 1:2+ | 1:2+ | 1:2+ |  |
| 169 | 1 | 2 | 2 | 4 | 1 | 1:16+ | -4 | 1:8+ | -1 | 1:8+ | 1:4+ | 1:1+ |  |
| 170 | 0 | 1 | 5 | 1 | 1 | 1:8+ | -3 | N | -4 | N |  |  | ≤3M- |
| 171 | 0 | 1 | 4 | 2 | 1 | 1:64+ | -6 | 1:16+ | -2 | 1:2+ |  |  | 13~24M- |
| 172 | 0 | 2 | 1 | 2 | 1 | 1:16+ | -4 | 1:4+ | -2 | 1:2+ |  |  | 7~12M- |
| 173 | 0 | 1 | 1 | 1 | 1 | 1:1+ | 0 | N | -1 | N |  |  | ≤3M- |
| 174 | 0 | 1 | 2 | 2 | 1 | 1:8+ | -3 | N | -4 | N |  |  | ≤3M- |
| 175 | 1 | 1 | 5 | 4 | 3 | 1:8+ | -3 | 1:16+ | 1 | 1:16+ | 1:4+ | ≥1:16+ |  |
| 176 | 0 | 2 | 2 | 4 | 1 | 1:8+ | -3 | 1:2+ | -2 | 1:1+ |  |  | 7~12M- |
| 177 | 0 | 2 | 2 | 1 | 1 | 1:32+ | -5 | 1:4+ | -3 | N |  |  | 4~6M- |
| 178 | 0 | 1 | 1 | 2 | 1 | 1:2+ | -1 | 1:1+ | -1 | 1:16+ |  |  | 13~24M- |
| 179 | 0 | 1 | 2 | 4 | 1 | 1:64+ | -6 | 1:16+ | -2 | 1:4+ |  |  | ＞24M- |
| 180 | 0 | 2 | 1 | 2 | 1 | 1:32+ | -5 | 1:4+ | -3 | 1:4+ |  |  | 13~24M- |
| 181 | 0 | 1 | 4 | 1 | 2 | 1:8+ | -3 | 1:2+ | -2 | 1:2+ |  |  | 7~12M- |
| 182 | 0 | 1 | 2 | 4 | 3 | 1:4+ | -2 | 1:2+ | -1 | 1:1+ |  |  | ＞24M- |
| 183 | 0 | 1 | 3 | 2 | 3 | 1:32+ | -5 | 1:1+ | -5 | 1:1+ |  |  | ＞24M- |
| 184 | 0 | 1 | 3 | 1 | 3 | 1:4+ | -2 | 1:2+ | -1 | 1:2+ |  |  | 7~12M- |
| 185 | 0 | 1 | 3 | 2 | 1 | 1:32+ | -5 | 1:4+ | -3 | 1:2+ |  |  | ＞24M- |
| 186 | 1 | 2 | 2 | 4 | 1 | 1:32+ | -5 | 1:8+ | -2 | 1:8+ | 1:4+ | 1:4+ |  |
| 187 | 0 | 1 | 4 | 1 | 1 | 1:32+ | -5 | N | -6 | N |  |  | ≤3M- |
| 188 | 1 | 2 | 6 | 2 | 1 | 1:256+ | -8 | 1:256+ | 0 | 1:64+ | 1:64+ | ≥1:16+ |  |
| 189 | 0 | 2 | 3 | 4 | 1 | 1:4+ | -2 | N | -3 | N |  |  | ≤3M- |
| 190 | 0 | 1 | 2 | 2 | 1 | 1:64+ | -6 | 1:2+ | -5 | N |  |  | 4~6M- |
| 191 | 0 | 2 | 5 | 1 | 1 | 1:16+ | -4 | 1:4+ | -2 | 1:4+ |  |  | 7~12M- |
| 192 | 0 | 1 | 2 | 1 | 1 | 1:2+ | -1 | N | -2 | N |  |  | ≤3M- |
| 193 | 0 | 2 | 3 | 2 | 1 | 1:64+ | -6 | 1:8+ | -3 | 1:2+ |  |  | 13~24M- |
| 194 | 0 | 1 | 3 | 2 | 1 | 1:32+ | -5 | 1:4+ | -3 | 1:2+ |  |  | 13~24M- |
| 195 | 0 | 2 | 1 | 2 | 1 | 1:32+ | -5 | 1:4+ | -3 | N |  |  | 4~6M- |
| 196 | 0 | 1 | 3 | 1 | 1 | 1:1+ | 0 | N | -1 | N |  |  | ≤3M- |
| 197 | 1 | 1 | 2 | 4 | 1 | 1:16+ | -4 | 1:8+ | -1 | 1:8+ | 1:4+ | 1:1+ |  |
| 198 | 0 | 1 | 3 | 1 | 1 | 1:64+ | -6 | 1:4+ | -4 | N |  |  | 4~6M- |
| 199 | 0 | 2 | 2 | 1 | 1 | 1:16+ | -4 | N | -5 | N |  |  | ≤3M- |
| 200 | 0 | 1 | 2 | 2 | 1 | 1:16+ | -4 | 1:4+ | -2 | N |  |  | 4~6M- |
| 201 | 0 | 1 | 1 | 2 | 1 | 1:64+ | -6 | 1:2+ | -5 | 1:1+ |  |  | ＞24M- |
| 202 | 0 | 2 | 1 | 2 | 1 | 1:16+ | -4 | N | -5 | N |  |  | ≤3M- |
| 203 | 0 | 1 | 2 | 4 | 1 | 1:16+ | -4 | N | -5 | N |  |  | ≤3M- |
| 204 | 1 | 1 | 4 | 4 | 2 | 1:16+ | -4 | 1:16+ | 0 | 1:8+ | 1:8+ | 1:8+ |  |
| 205 | 0 | 2 | 2 | 1 | 1 | 1:16+ | -4 | N | -5 | N |  |  | ≤3M- |
| 206 | 0 | 1 | 3 | 2 | 1 | 1:64+ | -6 | 1:16+ | -2 | 1:4+ |  |  | ＞24M- |
| 207 | 0 | 1 | 4 | 2 | 3 | 1:16+ | -4 | 1:1+ | -4 | 1:2+ |  |  | 13~24M- |
| 208 | 0 | 1 | 2 | 4 | 3 | 1:1+ | 0 | 1:1+ | 0 | 1:1+ |  |  | 7~12M- |
| 209 | 0 | 1 | 4 | 4 | 3 | 1:2+ | -1 | N | -2 | N |  |  | ≤3M- |
| 210 | 0 | 1 | 4 | 1 | 1 | 1:8+ | -3 | 1:8+ | 0 | 1:8+ |  |  | 7~12M- |
| 211 | 1 | 1 | 4 | 2 | 1 | 1:32+ | -5 | 1:2+ | -4 | 1:1+ | 1:1+ | 1:1+ |  |
| 212 | 0 | 2 | 2 | 2 | 1 | 1:16+ | -4 | 1:4+ | -2 | N |  |  | 4~6M- |
| 213 | 1 | 2 | 2 | 2 | 1 | 1:32+ | -5 | 1:8+ | -2 | 1:8+ | 1:4+ | 1:4+ |  |
| 214 | 1 | 2 | 5 | 2 | 1 | 1:64+ | -6 | 1:8+ | -3 | 1:8+ | 1:8+ | 1:8+ |  |
| 215 | 0 | 2 | 1 | 2 | 1 | 1:32+ | -5 | 1:2+ | -4 | N |  |  | 4~6M- |
| 216 | 1 | 2 | 2 | 2 | 1 | 1:32+ | -5 | 1:2+ | -4 | 1:8+ | 1:2+ | 1:1+ |  |
| 217 | 0 | 1 | 3 | 4 | 3 | 1:2+ | -1 | N | -2 | N |  |  | ≤3M- |
| 218 | 1 | 2 | 2 | 2 | 1 | 1:8+ | -3 | 1:4+ | -1 | 1:4+ | 1:4+ | 1:4+ |  |
| 219 | 0 | 1 | 5 | 1 | 1 | 1:16+ | -4 | N | -5 | N |  |  | ≤3M- |
| 220 | 0 | 2 | 4 | 2 | 1 | 1:32+ | -5 | 1:2+ | -4 | N |  |  | 4~6M- |
| 221 | 0 | 2 | 2 | 2 | 1 | 1:64+ | -6 | 1:4+ | -4 | N |  |  | 4~6M- |
| 222 | 1 | 1 | 4 | 4 | 1 | 1:16+ | -4 | 1:8+ | -1 | 1:8+ | 1:4+ | 1:4+ |  |
| 223 | 1 | 1 | 4 | 2 | 1 | 1:32+ | -5 | 1:2+ | -4 | 1:8+ | 1:4+ | 1:2+ |  |
| 224 | 0 | 1 | 4 | 4 | 1 | 1:64+ | -6 | 1:2+ | -5 | 1:2+ |  |  | ＞24M- |
| 225 | 0 | 2 | 3 | 1 | 2 | 1:32+ | -5 | 1:2+ | -4 | 1:2+ |  |  | 13~24M- |
| 226 | 0 | 1 | 5 | 2 | 1 | 1:4+ | -2 | 1:2+ | -1 | 1:2+ |  |  | 7~12M- |
| 227 | 1 | 2 | 2 | 2 | 1 | 1:64+ | -6 | 1:64+ | 0 | 1:32+ | 1:16+ | 1:8+ |  |
| 228 | 1 | 1 | 3 | 4 | 3 | 1:32+ | -5 | 1:16+ | -1 | 1:16+ | 1:8+ | 1:4+ |  |
| 229 | 1 | 2 | 3 | 4 | 1 | 1:32+ | -5 | 1:8+ | -2 | 1:16+ | 1:4+ | 1:4+ |  |
| 230 | 0 | 1 | 3 | 1 | 3 | 1:2+ | -1 | 1:1+ | -1 | N |  |  | 4~6M- |
| 231 | 0 | 1 | 3 | 4 | 1 | 1:8+ | -3 | 1:4+ | -1 | 1:1+ |  |  | 13~24M- |
| 232 | 1 | 2 | 2 | 4 | 1 | 1:16+ | -4 | 1:8+ | -1 | 1:8+ | 1:4+ | 1:4+ |  |
| 233 | 1 | 1 | 4 | 2 | 1 | 1:64+ | -6 | 1:32+ | -1 | 1:8+ | 1:16+ | 1:8+ |  |
| 234 | 0 | 2 | 6 | 2 | 1 | 1:128+ | -7 | 1:16+ | -3 | N |  |  | 4~6M- |
| 235 | 0 | 2 | 2 | 1 | 2 | 1:32+ | -5 | 1:16+ | -1 | N |  |  | 4~6M- |
| 236 | 0 | 1 | 4 | 1 | 1 | 1:8+ | -3 | N | -4 | N |  |  | ≤3M- |
| 237 | 1 | 1 | 4 | 4 | 1 | 1:16+ | -4 | 1:16+ | 0 | 1:4+ | 1:4+ | 1:4+ |  |
| 238 | 0 | 2 | 5 | 2 | 1 | 1:32+ | -5 | 1:32+ | 0 | 1:4+ |  |  | 7~12M- |
| 239 | 1 | 1 | 3 | 4 | 1 | 1:64+ | -6 | 1:16+ | -2 | 1:4+ | 1:2+ | 1:4+ |  |
| 240 | 0 | 2 | 2 | 2 | 1 | 1:32+ | -5 | 1:2+ | -4 | 1:2+ |  |  | 13~24M- |
| 241 | 0 | 1 | 1 | 2 | 1 | 1:32+ | -5 | 1:1+ | -5 | 1:1+ |  |  | 7~12M- |
| 242 | 1 | 2 | 2 | 4 | 1 | 1:32+ | -5 | 1:2+ | -4 | 1:8+ | 1:8+ | 1:4+ |  |
| 243 | 0 | 1 | 3 | 2 | 1 | 1:64+ | -6 | 1:1+ | -6 | N |  |  | 4~6M- |
| 244 | 0 | 2 | 4 | 4 | 3 | 1:1+ | 0 | 1:1+ | 0 | 1:1+ |  |  | 7~12M- |
| 245 | 1 | 2 | 1 | 2 | 1 | 1:8+ | -3 | 1:4+ | -1 | 1:1+ | 1:1+ | 1:1+ |  |
| 246 | 0 | 2 | 5 | 4 | 1 | 1:1+ | 0 | N | -1 | N |  |  | ≤3M- |
| 247 | 0 | 1 | 3 | 1 | 3 | 1:4+ | -2 | N | -3 | N |  |  | ≤3M- |
| 248 | 0 | 1 | 3 | 2 | 1 | 1:16+ | -4 | 1:2+ | -3 | N |  |  | 4~6M- |
| 249 | 0 | 1 | 3 | 2 | 2 | 1:64+ | -6 | 1:2+ | -5 | N |  |  | 4~6M- |
| 250 | 0 | 1 | 4 | 2 | 3 | 1:16+ | -4 | 1:4+ | -2 | 1:4+ |  |  | 7~12M- |
| 251 | 0 | 1 | 3 | 2 | 1 | 1:32+ | -5 | 1:8+ | -2 | N |  |  | 4~6M- |
| 252 | 0 | 1 | 6 | 4 | 1 | 1:1+ | 0 | N | -1 | N |  |  | ≤3M- |
| 253 | 1 | 1 | 2 | 4 | 1 | 1:2+ | -1 | 1:2+ | 0 | 1:2+ | 1:2+ | 1:1+ |  |
| 254 | 1 | 1 | 4 | 2 | 1 | 1:64+ | -6 | 1:16+ | -2 | 1:16+ | 1:8+ | 1:8+ |  |
| 255 | 0 | 1 | 6 | 1 | 1 | 1:32+ | -5 | 1:16+ | -1 | 1:2+ |  |  | 7~12M- |
| 256 | 0 | 2 | 3 | 2 | 2 | 1:32+ | -5 | 1:8+ | -2 | N |  |  | 4~6M- |
| 257 | 1 | 2 | 2 | 2 | 2 | 1:4+ | -2 | 1:4+ | 0 | 1:4+ | 1:4+ | 1:4+ |  |
| 258 | 1 | 1 | 4 | 4 | 1 | 1:64+ | -6 | 1:16+ | -2 | 1:16+ | 1:4+ | 1:4+ |  |
| 259 | 1 | 1 | 1 | 4 | 1 | 1:64+ | -6 | 1:16+ | -2 | 1:8+ | 1:16+ | 1:4+ |  |
| 260 | 0 | 1 | 2 | 2 | 1 | 1:16+ | -4 | 1:2+ | -3 | 1:16+ |  |  | 7~12M- |
| 261 | 0 | 1 | 2 | 2 | 1 | 1:16+ | -4 | 1:4+ | -2 | 1:1+ |  |  | 13~24M- |
| 262 | 0 | 1 | 2 | 2 | 1 | 1:2+ | -1 | 1:1+ | -1 | N |  |  | 4~6M- |
| 263 | 0 | 2 | 2 | 2 | 1 | 1:16+ | -4 | N | -5 | N |  |  | ≤3M- |
| 264 | 1 | 2 | 5 | 4 | 3 | 1:32+ | -5 | 1:32+ | 0 | 1:32+ | 1:32+ | ≥1:16+ |  |
| 265 | 0 | 1 | 5 | 1 | 1 | 1:32+ | -5 | 1:2+ | -4 | N |  |  | 4~6M- |
| 266 | 0 | 1 | 2 | 1 | 1 | 1:64+ | -6 | 1:16+ | -2 | 1:1+ |  |  | 7~12M- |
| 267 | 0 | 1 | 2 | 1 | 1 | 1:2+ | -1 | 1:1+ | -1 | N |  |  | 4~6M- |
| 268 | 0 | 1 | 3 | 2 | 1 | 1:16+ | -4 | 1:4+ | -2 | 1:1+ |  |  | 7~12M- |
| 269 | 0 | 1 | 2 | 2 | 1 | 1:16+ | -4 | N | -5 | N |  |  | ≤3M- |
| 270 | 0 | 1 | 5 | 1 | 1 | 1:64+ | -6 | 1:16+ | -2 | N |  |  | 4~6M- |
| 271 | 0 | 2 | 4 | 2 | 1 | 1:16+ | -4 | 1:2+ | -3 | N |  |  | 4~6M- |
| 272 | 0 | 1 | 4 | 2 | 1 | 1:64+ | -6 | 1:16+ | -2 | 1:16+ |  |  | 7~12M- |
| 273 | 0 | 2 | 3 | 2 | 1 | 1:256+ | -8 | 1:16+ | -4 | 1:8+ |  |  | 7~12M- |
| 274 | 0 | 2 | 4 | 4 | 3 | 1:1+ | 0 | 1:1+ | 0 | 1:1+ |  |  | ＞24M- |
| 275 | 1 | 1 | 4 | 2 | 1 | 1:64+ | -6 | 1:16+ | -2 | 1:16+ | 1:16+ | ≥1:16+ |  |
| 276 | 0 | 1 | 1 | 1 | 1 | 1:16+ | -4 | 1:2+ | -3 | N |  |  | 4~6M- |
| 277 | 1 | 1 | 5 | 4 | 1 | 1:32+ | -5 | 1:16+ | -1 | 1:16+ | 1:16+ | 1:2+ |  |
| 278 | 0 | 1 | 3 | 2 | 3 | 1:2+ | -1 | 1:1+ | -1 | 1:1+ |  |  | 7~12M- |
| 279 | 0 | 2 | 1 | 2 | 3 | 1:32+ | -5 | 1:1+ | -5 | 1:1+ |  |  | 13~24M- |
| 280 | 0 | 1 | 2 | 2 | 1 | 1:64+ | -6 | 1:8+ | -3 | 1:1+ |  |  | 13~24M- |
| 281 | 1 | 1 | 4 | 4 | 2 | 1:16+ | -4 | 1:16+ | 0 | 1:8+ | 1:8+ | 1:8+ |  |
| 282 | 0 | 1 | 1 | 4 | 1 | 1:16+ | -4 | 1:1+ | -4 | 1:16+ |  |  | ＞24M- |
| 283 | 0 | 1 | 1 | 1 | 1 | 1:2+ | -1 | N | -2 | N |  |  | ≤3M- |
| 284 | 1 | 2 | 6 | 4 | 3 | 1:4+ | -2 | 1:16+ | 2 | 1:4+ | 1:4+ | 1:4+ |  |
| 285 | 0 | 1 | 5 | 1 | 2 | 1:1+ | 0 | 1:1+ | 0 | 1:1+ |  |  | 7~12M- |
| 286 | 0 | 1 | 4 | 1 | 3 | 1:8+ | -3 | 1:2+ | -2 | N |  |  | 4~6M- |
| 287 | 0 | 1 | 3 | 2 | 1 | 1:8+ | -3 | 1:2+ | -2 | N |  |  | 4~6M- |
| 288 | 1 | 1 | 4 | 4 | 1 | 1:4+ | -2 | 1:8+ | 1 | 1:8+ | 1:4+ | 1:4+ |  |
| 289 | 1 | 1 | 3 | 4 | 1 | 1:16+ | -4 | 1:8+ | -1 | 1:8+ | 1:8+ | 1:2+ |  |
| 290 | 0 | 1 | 4 | 2 | 1 | 1:32+ | -5 | 1:2+ | -4 | N |  |  | 4~6M- |
| 291 | 0 | 1 | 2 | 1 | 1 | 1:2+ | -1 | N | -2 | N |  |  | ≤3M- |
| 292 | 1 | 1 | 1 | 2 | 3 | 1:32+ | -5 | 1:1+ | -5 | 1:1+ | 1:1+ | 1:1+ |  |
| 293 | 0 | 2 | 5 | 2 | 1 | 1:16+ | -4 | 1:4+ | -2 | N |  |  | 4~6M- |
| 294 | 1 | 1 | 2 | 4 | 1 | 1:8+ | -3 | 1:1+ | -3 | 1:1+ | 1:1+ | 1:1+ |  |
| 295 | 0 | 1 | 3 | 2 | 1 | 1:2+ | -1 | N | -2 | N |  |  | ≤3M- |
| 296 | 0 | 1 | 2 | 1 | 1 | 1:4+ | -2 | N | -3 | N |  |  | ≤3M- |
| 297 | 0 | 1 | 2 | 2 | 2 | 1:16+ | -4 | N | -5 | N |  |  | ≤3M- |
| 298 | 0 | 1 | 3 | 2 | 1 | 1:32+ | -5 | 1:32+ | 0 | 1:32+ |  |  | 7~12M- |
| 299 | 0 | 1 | 4 | 1 | 1 | 1:2+ | -1 | 1:1+ | -1 | N |  |  | 4~6M- |
| 300 | 1 | 1 | 2 | 4 | 1 | 1:8+ | -3 | 1:4+ | -1 | 1:2+ | 1:4+ | 1:4+ |  |
| 301 | 1 | 1 | 3 | 2 | 1 | 1:8+ | -3 | 1:4+ | -1 | 1:4+ | 1:4+ | 1:4+ |  |
| 302 | 1 | 1 | 3 | 2 | 1 | 1:16+ | -4 | 1:1+ | -4 | 1:16+ | 1:2+ | 1:2+ |  |
| 303 | 1 | 2 | 3 | 4 | 2 | 1:128+ | -7 | 1:4+ | -5 | 1:1+ | 1:1+ | 1:1+ |  |
| 304 | 1 | 1 | 5 | 4 | 1 | 1:16+ | -4 | 1:2+ | -3 | 1:2+ | 1:2+ | 1:2+ |  |
| 305 | 1 | 2 | 2 | 2 | 1 | 1:128+ | -7 | 1:8+ | -4 | 1:4+ | 1:1+ | 1:1+ |  |
| 306 | 0 | 1 | 2 | 1 | 1 | 1:2+ | -1 | N | -2 | N |  |  | ≤3M- |
| 307 | 0 | 1 | 3 | 2 | 1 | 1:32+ | -5 | 1:2+ | -4 | 1:2+ |  |  | 7~12M- |
| 308 | 0 | 1 | 3 | 2 | 1 | 1:64+ | -6 | 1:1+ | -6 | 1:1+ |  |  | 7~12M- |
| 309 | 0 | 1 | 4 | 4 | 1 | 1:32+ | -5 | 1:8+ | -2 | N |  |  | 4~6M- |
| 310 | 0 | 1 | 3 | 2 | 1 | 1:4+ | -2 | 1:1+ | -2 | 1:1+ |  |  | 7~12M- |
| 311 | 1 | 1 | 2 | 4 | 2 | 1:16+ | -4 | 1:8+ | -1 | 1:8+ | 1:8+ | 1:8+ |  |
| 312 | 0 | 1 | 5 | 2 | 1 | 1:32+ | -5 | 1:1+ | -5 | N |  |  | 4~6M- |
| 313 | 1 | 1 | 4 | 4 | 1 | 1:4+ | -2 | 1:2+ | -1 | 1:4+ | 1:4+ | 1:4+ |  |
| 314 | 0 | 2 | 5 | 2 | 1 | 1:64+ | -6 | 1:4+ | -4 | N |  |  | 4~6M- |
| 315 | 0 | 2 | 4 | 4 | 2 | 1:1+ | 0 | N | -1 | N |  |  | ≤3M- |
| 316 | 0 | 1 | 2 | 4 | 1 | 1:2+ | -1 | N | -2 | N |  |  | ≤3M- |
| 317 | 0 | 1 | 3 | 4 | 1 | 1:8+ | -3 | 1:8+ | 0 | N |  |  | 4~6M- |
| 318 | 0 | 2 | 2 | 1 | 1 | 1:2+ | -1 | N | -2 | N |  |  | ≤3M- |
| 319 | 1 | 2 | 6 | 4 | 1 | 1:2+ | -1 | 1:4+ | 1 | 1:2+ | 1:2+ | 1:2+ |  |
| 320 | 1 | 1 | 3 | 4 | 1 | 1:64+ | -6 | 1:32+ | -1 | 1:16+ | 1:16+ | ≥1:16+ |  |
| 321 | 0 | 1 | 4 | 4 | 1 | 1:8+ | -3 | 1:2+ | -2 | N |  |  | 4~6M- |
| 322 | 0 | 1 | 4 | 1 | 1 | 1:1+ | 0 | N | -1 | N |  |  | ≤3M- |
| 323 | 0 | 2 | 2 | 2 | 1 | 1:32+ | -5 | N | -6 | N |  |  | ≤3M- |
| 324 | 1 | 2 | 5 | 4 | 1 | 1:32+ | -5 | 1:8+ | -2 | 1:8+ | 1:8+ | 1:8+ |  |
| 325 | 0 | 1 | 3 | 4 | 1 | 1:32+ | -5 | N | -6 | N |  |  | ≤3M- |
| 326 | 0 | 1 | 2 | 1 | 1 | 1:2+ | -1 | 1:1+ | -1 | N |  |  | 4~6M- |
| 327 | 1 | 1 | 2 | 2 | 1 | 1:64+ | -6 | 1:4+ | -4 | 1:4+ | 1:8+ | 1:2+ |  |
| 328 | 0 | 2 | 4 | 2 | 1 | 1:16+ | -4 | 1:4+ | -2 | 1:4+ |  |  | 7~12M- |
| 329 | 0 | 1 | 2 | 2 | 1 | 1:8+ | -3 | 1:2+ | -2 | N |  |  | 4~6M- |
| 330 | 1 | 2 | 2 | 4 | 2 | 1:1+ | 0 | 1:1+ | 0 | 1:1+ | 1:1+ | 1:1+ |  |
| 331 | 0 | 1 | 4 | 4 | 1 | 1:1+ | 0 | N | -1 | N |  |  | ≤3M- |
| 332 | 0 | 1 | 3 | 1 | 1 | 1:2+ | -1 | 1:1+ | -1 | N |  |  | 4~6M- |
| 333 | 1 | 1 | 3 | 2 | 1 | 1:32+ | -5 | 1:8+ | -2 | 1:4+ | 1:4+ | 1:4+ |  |
| 334 | 0 | 2 | 2 | 4 | 1 | 1:1+ | 0 | 1:1+ | 0 | N |  |  | 4~6M- |
| 335 | 0 | 1 | 3 | 1 | 1 | 1:2+ | -1 | N | -2 | N |  |  | ≤3M- |
| 336 | 0 | 1 | 2 | 2 | 1 | 1:16+ | -4 | 1:2+ | -3 | N |  |  | 4~6M- |
| 337 | 0 | 2 | 2 | 2 | 1 | 1:64+ | -6 | 1:8+ | -3 | 1:2+ |  |  | 7~12M- |
| 338 | 0 | 1 | 5 | 2 | 1 | 1:32+ | -5 | 1:8+ | -2 | N |  |  | 4~6M- |
| 339 | 0 | 1 | 2 | 2 | 1 | 1:8+ | -3 | N | -4 | N |  |  | ≤3M- |
| 340 | 1 | 2 | 6 | 4 | 2 | 1:2+ | -1 | 1:2+ | 0 | 1:1+ | 1:1+ | 1:1+ |  |
| 341 | 1 | 1 | 2 | 2 | 1 | 1:32+ | -5 | 1:4+ | -3 | 1:1+ | 1:8+ | 1:2+ |  |
| 342 | 1 | 2 | 2 | 2 | 1 | 1:4+ | -2 | 1:4+ | 0 | 1:4+ | 1:4+ | 1:4+ |  |
| 343 | 1 | 2 | 2 | 2 | 1 | 1:16+ | -4 | 1:8+ | -1 | 1:2+ | 1:4+ | 1:2+ |  |
| 344 | 0 | 1 | 3 | 4 | 1 | 1:32+ | -5 | 1:4+ | -3 | N |  |  | 4~6M- |
| 345 | 1 | 2 | 5 | 4 | 1 | 1:2+ | -1 | 1:2+ | 0 | 1:2+ | 1:2+ | 1:2+ |  |
| 346 | 0 | 1 | 2 | 2 | 1 | 1:32+ | -5 | 1:1+ | -5 | N |  |  | 4~6M- |
| 347 | 0 | 1 | 3 | 1 | 1 | 1:2+ | -1 | N | -2 | N |  |  | ≤3M- |
| 348 | 0 | 2 | 3 | 2 | 1 | 1:32+ | -5 | 1:1+ | -5 | N |  |  | 4~6M- |
| 349 | 1 | 2 | 2 | 2 | 1 | 1:32+ | -5 | 1:4+ | -3 | 1:4+ | 1:4+ | 1:4+ |  |
| 350 | 1 | 2 | 5 | 2 | 1 | 1:64+ | -6 | 1:16+ | -2 | 1:16+ | 1:8+ | 1:4+ |  |
| 351 | 0 | 1 | 2 | 4 | 1 | 1:8+ | -3 | N | -4 | N |  |  | ≤3M- |
| 352 | 0 | 1 | 6 | 4 | 1 | 1:1+ | 0 | N | -1 | N |  |  | ≤3M- |
| 353 | 1 | 1 | 4 | 2 | 2 | 1:64+ | -6 | 1:16+ | -2 | 1:4+ | 1:2+ | 1:2+ |  |
| 354 | 1 | 1 | 4 | 4 | 1 | 1:4+ | -2 | 1:4+ | 0 | 1:4+ | 1:4+ | 1:1+ |  |
| 355 | 1 | 1 | 2 | 1 | 1 | 1:16+ | -4 | 1:4+ | -2 | 1:1+ | 1:1+ | 1:1+ |  |
| 356 | 0 | 1 | 2 | 4 | 1 | 1:32+ | -5 | 1:1+ | -5 | 1:1+ |  |  | 7~12M- |
| 357 | 0 | 1 | 2 | 2 | 1 | 1:16+ | -4 | 1:2+ | -3 | N |  |  | 4~6M- |
| 358 | 0 | 2 | 2 | 2 | 1 | 1:16+ | -4 | N | -5 | N |  |  | ≤3M- |
| 359 | 0 | 1 | 2 | 4 | 1 | 1:8+ | -3 | 1:1+ | -3 | N |  |  | 4~6M- |
| 360 | 0 | 1 | 3 | 2 | 1 | 1:1+ | 0 | N | -1 | N |  |  | ≤3M- |
| 361 | 0 | 1 | 2 | 4 | 1 | 1:1+ | 0 | N | -1 | N |  |  | ≤3M- |
| 362 | 0 | 2 | 3 | 2 | 1 | 1:16+ | -4 | 1:1+ | -4 | N |  |  | 4~6M- |
| 363 | 0 | 1 | 3 | 2 | 1 | 1:16+ | -4 | 1:1+ | -4 | 1:1+ |  |  | 7~12M- |
| 364 | 0 | 1 | 2 | 2 | 1 | 1:16+ | -4 | 1:1+ | -4 | N |  |  | 4~6M- |
| 365 | 0 | 1 | 1 | 2 | 1 | 1:32+ | -5 | 1:1+ | -5 | N |  |  | 4~6M- |
| 366 | 0 | 1 | 6 | 4 | 1 | 1:1+ | 0 | 1:1+ | 0 | N |  |  | 4~6M- |
| 367 | 0 | 1 | 3 | 2 | 1 | 1:32+ | -5 | 1:2+ | -4 | 1:2+ |  |  | 7~12M- |
| 368 | 0 | 2 | 2 | 1 | 1 | 1:1+ | 0 | N | -1 | N |  |  | ≤3M- |
| 369 | 0 | 1 | 2 | 1 | 1 | 1:1+ | 0 | N | -1 | N |  |  | ≤3M- |
| 370 | 0 | 1 | 3 | 1 | 1 | 1:4+ | -2 | N | -3 | N |  |  | ≤3M- |
| 371 | 0 | 1 | 5 | 1 | 1 | 1:2+ | -1 | N | -2 | N |  |  | ≤3M- |
| 372 | 0 | 1 | 4 | 4 | 1 | 1:1+ | 0 | N | -1 | N |  |  | ≤3M- |
| 373 | 0 | 1 | 3 | 4 | 1 | 1:1+ | 0 | N | -1 | N |  |  | ≤3M- |
